# The CagRS Two-Component System Regulates Clavulanic Acid Metabolism via Multiple Pathways in *Streptomyces clavuligerus* F613-1

**DOI:** 10.3389/fmicb.2019.00244

**Published:** 2019-02-14

**Authors:** Jiafang Fu, Ronghuo Qin, Gongli Zong, Cheng Liu, Ni Kang, Chuanqing Zhong, Guangxiang Cao

**Affiliations:** ^1^Shandong Medicinal Biotechnology Center, Shandong Academy of Medical Sciences, Jinan, China; ^2^School of Municipal and Environmental Engineering, Shandong Jianzhu University, Jinan, China

**Keywords:** *Streptomyces clavuligerus*, clavulanic acid, CagRS, arginine, glyceraldehyde 3-phosphate, primary metabolism

## Abstract

*Streptomyces clavuligerus* F613-1 produces a clinically important β-lactamase inhibitor, clavulanic acid (CA). Although the biosynthesis pathway of CA has essentially been elucidated, the global regulatory mechanisms of CA biosynthesis remain unclear. The paired genes *cagS* and *cagR*, which are annotated, respectively, as *orf22* and *orf23* in *S. clavuligerus* ATCC 27064, encode a bacterial two-component regulatory system (TCS) and were found next to the CA biosynthetic gene cluster of *S. clavuligerus* F613-1. To further elucidate the regulatory mechanism of CA biosynthesis, the CagRS TCS was deleted from *S. clavuligerus* F613-1. Deletion of *cagRS* resulted in decreased production of CA, but the strain phenotype was not otherwise affected. Both transcriptome and ChIP-seq data revealed that, in addition to CA biosynthesis, the CagRS TCS mainly regulates genes involved in primary metabolism, such as glyceraldehyde 3-phosphate (G3P) metabolism and arginine biosynthesis. Notably, both G3P and arginine are precursors of CA. Electrophoretic mobility shift assays demonstrated that the response regulator CagR could bind to the intergenic regions of *argG, argC, oat1, oat2, ceaS1*, and *claR in vitro*, suggesting that CagR can directly regulate genes involved in arginine and CA biosynthesis. This study indicated that CagRS is a pleiotropic regulator that can directly affect the biosynthesis of CA and indirectly affect CA production by regulating the metabolism of arginine and G3P. Our findings provide new insights into the regulation of CA biosynthetic pathways and provide an innovative approach for future metabolic engineering efforts for CA production in *S. clavuligerus*.

## Introduction

Clavulanic acid (CA), a broad-spectrum inhibitor of beta-lactamase, is widely used clinically in combination with penicillin and cephalosporin, as it can effectively improve the antibacterial effect of β-lactam antibiotics (Saudagar et al., 2008). The unique three-dimensional structure (3*R*, 5*R*) of CA enables it to irreversibly combine with serine hydroxyl from the active center of β-lactamase, thereby inactivating this β-lactam resistance mechanism (Liras and Rodriguez-Garcia, 2000). *Streptomyces clavuligerus* was first isolated and screened for the production of CA (Reading and Cole, 1977), and although *S. jumonjinensis* and *S. katsurahamanus* were also found to produce CA, *S. clavuligerus* is the major production strain for CA (Brown et al., 1976; Jensen and Paradkar, 1999). In addition to CA, *S. clavuligerus* also produces the β-lactam antibiotic cephamycin C and several compounds with a clavam structure and (3*S*, 5*S*) stereochemistry; these compounds are considered to be the main by-products of CA fermentation.

**FIGURE 1:**
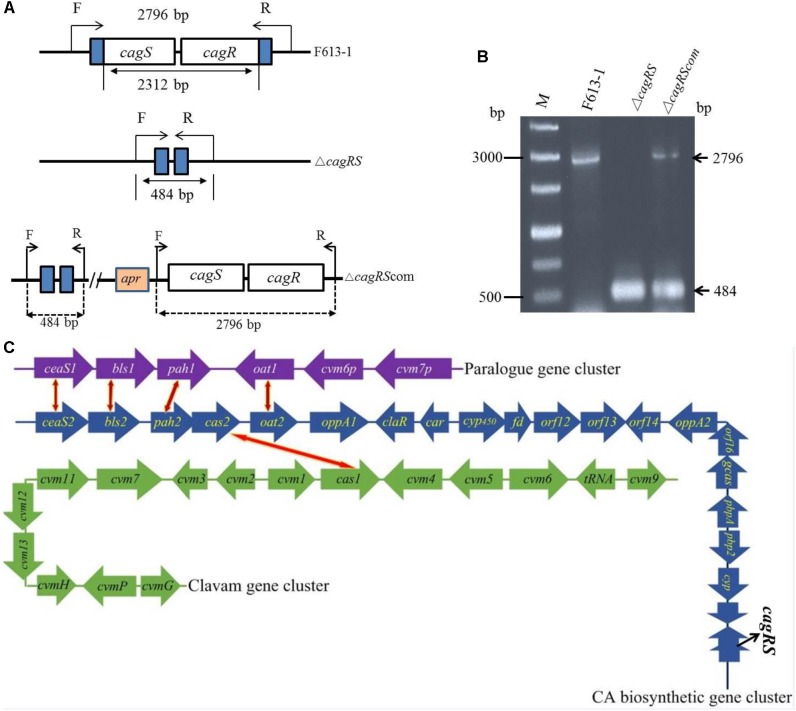
Verification of the *cagRS* deletion strain Δ*cagRS*. **(A)** Schematic diagram of the construction of the *cagRS* mutant and complemented strains. F and R represent forward and reverse primers, respectively. **(B)** PCR verification of the Δ*cagRS* mutant. M: DNA marker. The F/R primers are located at the flanking regions of the TCS. **(C)** Schematic diagram of CA biosynthesis-related gene clusters in F613-1. The red and yellow bidirectional arrows indicate homologous genes. The CA biosynthetic gene cluster (blue arrows) includes five operons: *ceaS2*-*bls2, pah2*-*cas2, cyp450*-*fd, orf12*-*orf13* and *gcaS*-*orf16*-*oppA2.*

Three clusters of genes involved in CA biosynthesis have been isolated in *S. clavuligerus*: the CA biosynthetic gene cluster, the clavam gene cluster, and the paralog gene cluster (Figure 1C). Both the CA biosynthetic gene cluster and the clavam gene cluster are located on the chromosome of *S. clavuligerus*, whereas the paralog gene cluster is located on the pSCL4 plasmid (Jensen et al., 2000; Liras et al., 2008; Song et al., 2010). The CA biosynthetic gene cluster was initially isolated by hybridization with the *cas2* gene, encoding clavaminate synthase, and includes the six biosynthetic enzyme-encoding genes *bls2, pah*2, *ceaS2, cas2, car* (also known as *cad*) and *gcaS* (Baggaley et al., 1997; Arulanantham et al., 2006); two genes, *pbpA* and *pbp2*, encoding penicillin-binding proteins (Ishida et al., 2006); the *oat2* gene encoding ornithine acetyltransferase (Hodgson et al., 1995); two genes, *oppA1* and *oppA2*, encoding oligopeptide permeases (Lorenzana et al., 2004; Mackenzie et al., 2010); gene *claR*, which encodes a LysR-type regulatory protein (Liras and Rodriguez-Garcia, 2000); and the *cyp450*-*fd, orf12, orf13, orf14*, and *orf16* genes, which are required for CA biosynthesis but are of unknown function.

The clavam gene cluster was isolated using the *cas1* gene in *S. clavuligerus*, a duplicate gene encoding a clavaminate synthase isoenzyme, as probe (Mosher et al., 1999). This gene cluster contains genes *cvm1* to *cvm13, cvmG, cvmH, cvmP*, and *cas1*, although only *cas1* is required for CA biosynthesis (Liras et al., 2008). Additionally, although *cas1* is a homolog of *cas2* of the CA biosynthetic gene cluster, the two genes are regulated by different mechanisms (Paradkar and Jensen, 1995).

The paralog gene cluster contains the genes *ceaS1, bls1, pah1, oat1, cvm6p*, and *cvm7p* (Jensen et al., 2000, 2004a), and the first four of these genes may have been duplicated from genes of the CA biosynthetic gene cluster (Tahlan et al., 2004a,b). *ceaS1* is a homolog of *ceaS2*, with 73% similarity; *bls1* is a homolog of *bls2*, with 60% similarity; and *oat1* is a homolog of *oat2*, with 63% similarity. *pah1* encodes a protein that functions similarly to Pah2, and the two proteins have a sequence similarity of 72%. It has been reported that deletion of *pah1* resulted in significantly reduced production of CA as well as of 5*S*-clavam (Jensen et al., 2004b).

The CA biosynthesis is regulated by several mechanisms in *S. clavuligerus*, including the two pathway-specific regulatory factors ClaR and CcaR. The ClaR-encoding gene, *claR*, is located in the CA biosynthetic gene cluster, and studies have shown that ClaR can positively regulate CA biosynthesis (Paradkar et al., 1998; Perez-Redondo et al., 1998; Gomez-Escribano et al., 2006; Martinez-Burgo et al., 2015). The CcaR-encoding gene, *ccaR*, is located in the cephamycin C gene cluster, and CcaR is reported to positively regulate the expression of genes involved in the early stage of CA biosynthesis, such as *ceaS2, bls2, pah2*, and *cas2* (Santamarta et al., 2002, 2011; Alvarez-Alvarez et al., 2014). In addition, CcaR also binds the promoter of *claR* (Santamarta et al., 2011; Kurt et al., 2013), indicating that ClaR and CcaR may jointly form a regulatory system to regulate the biosynthesis of CA (Kurt et al., 2013; Kwong et al., 2013).

In addition to the CA pathway-specific regulatory factors, many other factors regulate CA biosynthesis. Deletion of the γ-butyrolactone receptor protein Brp can result in increased CA production, and Brp negatively regulates the biosynthesis of CA through inhibiting *ccaR* (Santamarta et al., 2005). BldG is an upstream regulatory factor that can regulate CA biosynthesis by regulating *ccaR* expression (Bignell et al., 2005), and the sigma factor encoded by *orf21* can bind to the *ccaR* promoter region and thereby also influence CA biosynthesis (Jnawali et al., 2011). In addition, CA biosynthesis was reported to be negatively regulated by *ccaR* and *claR* when amino acids are scarce (Gomez-Escribano et al., 2008).

Enhancement of CA production is a very important goal for the commercial pharmaceutical market. Generally, there are two main ways to increase CA production (Paradkar et al., 2001; Jnawali et al., 2010): (1) optimize the medium and conditions for CA fermentation, and (2) clarify and optimize the biosynthetic and regulatory mechanisms of CA production. The biosynthesis pathway of CA and its related by-product (clavam) has been partially elucidated (Liras et al., 2008). However, the mechanisms regulating CA biosynthesis have not been fully delineated, and there are no reports about the global regulation of CA biosynthesis. Therefore, in addition to manipulating genes encoding regulatory factors (such as *claR, ccaR*) and known essential biosynthetic enzymes (such as *bls2, pah*2, *ceaS2, cas2, car*, and *gcaS*), elucidation of the functions of other genes responsible for as yet unknown but essential roles in CA biosynthesis may suggest other ways to increase production of CA.

Two-component systems (TCSs) are normally organized as pairs on bacterial genomes, with co-transcription of the response regulator and histidine kinase genes. TCSs not only respond to changes in environmental factors, but in *Streptomyces*, also influence development and secondary metabolism such as antibiotic production (Mendes et al., 2007). *S. clavuligerus* F613-1 is an industrial CA producer strain, and we have previously reported the complete genome sequence of this strain (Cao et al., 2016). In this study, we identified TCS CagRS, which is annotated as orf22/orf23 in *S. clavuligerus* 27064 (Song et al., 2009) and which is close to the CA biosynthetic gene cluster in F613-1. We investigated the effects of TCS CagRS on CA production in *S. clavuligerus* F613-1, and our results provide insights into new approaches for improving CA yield.

## Materials and Methods

### Plasmids and Bacterial Strains

All strains and plasmids used in this study are listed in Supplementary Table S1. *S. clavuligerus* F613-1 is an industrial strain (Jin et al., 2015) and was used as the parental strain in this study. Cloning procedures were performed in *Escherichia coli* DH5a, protein expression was performed using *E. coli* BL21(DE3), and *E. coli* ET12567/pUZ8002 was used for intergeneric conjugative transfer of plasmid DNA into *S. clavuligerus* (Kieser et al., 2000).

### Primers

All primers used in the construction of the Δ*cagRS* deletion mutant strain and complemented strain, construction of a CagR-3 × FLAG-complemented *S. clavuligerus* strain, confirmation of conjugants, and in EMSAs and real-time PCR analysis are listed in Supplementary Table S2.

### Culture Conditions

*Escherichia coli* strains were grown at 37°C in LB medium or on solid LB plates supplemented with ampicillin (100 μg/mL), kanamycin (25 μg/mL), or chloramphenicol (25 μg/mL) when required.

Culturing of *S. clavuligerus* F613-1 and the strains derived from it was performed as described previously (Qin et al., 2017). *S. clavuligerus* transformants were cultured on MS plates (2.0% soybean powder, 2% glycerol, and 2.0% agar powder, pH 7.3) supplemented with nalidixic acid (25 mg/mL) and thiostrepton (15 mg/mL) to select pJTU1278-derived plasmids, and the disrupted mutants were selected with antibiotic-free MS solid media or with apramycin (15 mg/mL) to select pSET152-derived plasmids.

*Streptomyces clavuligerus* F613-1 and its derived strains were grown at 25°C with a relative humidity of 50-60% on BSCA plates (1.5% malt extract, 0.3% tryptone, 0.4% glucose, and 2.0% agar powder, pH 7.5) for 8 days for collection of spores. For bioassay analysis, F613-1 and derived strains were grown at 25°C with a relative humidity of 50-60% on BSCA plates for 3–9 days; on MSF plates (2.0% soybean powder, 2% mannitol, and 2.0% agar powder, pH 7.3) for 5–11 days; on MM solid media (0.05% L-asparagine, 0.05% dipotassium hydrogen phosphate, 0.02% MgSO_4_⋅7H_2_O, 0.001% FeSO_4_⋅7H_2_O, 1% dextrose monohydrate, and 2.0% agar powder, pH 7.2) for 9–15 days; or on ISP4 solid media (1% soluble starch, 0.1% dipotassium hydrogen phosphate, 0.1% MgSO_4_⋅7H_2_O, 0.1% NaCl, 0.2% ammonium sulfate, 0.2% CaCO_3_, 0.0001% FeSO_4_⋅7H_2_O, 0.0001% MnCl_2_⋅7H_2_O, and 2.0% agar powder, pH 7.2) for 7–13 days.

For the liquid-state fermentation, spores (2 × 10^6^) of the *S. clavuligerus* strains were inoculated into 100 mL SCZ seed medium [2.0% soybean powder, 1.2% maize starch, 0.5% yeast extract, 0.08% dipotassium hydrogen phosphate, and 1.1% (v/v) glycerol trioleate, pH 7.1), and then cultured at 25°C, with shaking on an orbital shaker at 200 rpm for 48 h to obtain seed cultures. Next, 5 mL seed cultures were transferred to 100 mL SCF fermentation medium (2.7% soybean powder, 2.2% soybean protein extract, 3.0% maltodextrin, 0.15% potassium chloride, 0.1% magnesium chloride hexahydrate, 0.2% dipotassium hydrogen phosphate, 0.04% calcium chloride dihydrate, 0.008% ferric chloride hexahydrate, 0.001% zinc chloride, 0.018% sodium chloride, and 4.2% MOPS, pH 7.1) supplemented with or without 1.6% (v/v) glycerol trioleate and grown at 25°C, with shaking on an orbital shaker at 200 rpm. For HPLC analysis of CA, 1 mL samples of fermentation liquid were collected at 24, 72, 120, 168, and 216 h and centrifuged at 5000 rpm to collect the supernatant. In addition, the biomass was measured before the analysis of CA production, using 1 g samples of fermentation liquid centrifuged at 5000 rpm to collect the mycelium.

### DNA Manipulation

Genomic DNA from *S. clavuligerus* was isolated using the Kirby mix procedure (Kieser et al., 2000). Plasmids were extracted from *E. coli* using plasmid mini-prep columns (BioTeke, China) according to the manufacturer’s protocol. Restriction endonuclease digestions of plasmid DNA were carried out according to the manufacturer’s recommendations. DNA ligation was performed using Solution I (TaKaRa, Japan) according to the manufacturer’s recommendations.

### Construction and Complementation of a *cagRS* Null Mutant

The *cagRS* genes were knocked out through homologous recombination using a strategy similar to that described (Gust et al., 2003; Zhang et al., 2014). The DNA fragment serving as the left arm of *cagRS* was amplified by PCR using primers cagRS L-F/R and *S. clavuligerus* F613-1 genomic DNA as template, and then the amplified PCR products were cloned into the general cloning vector pEasy-Blunt-Simple to obtain the recombinant plasmid pEBS-cagRS L. The DNA fragment serving as the right arm of *cagRS* was amplified by PCR using primers cagRS R-F/R, and then the amplified PCR products were cloned into the general cloning vector pEasy-Blunt-Simple to obtain the recombinant plasmid pEBS-cagRS R. After verification by DNA sequencing, both pEBS-cagRS L and pEBS-cagRS R were digested with *Hind*III and *Bam*HI, and the digested right arm of *cagRS* was cloned into the digested recombinant plasmid pEBS-cagRS L to obtain the recombinant plasmid pEBS-cagRS L/R. After verification by DNA sequencing, the *cagRS* L/R fragment was excised from pEBS-cagRS L/R using *Spe*I and *Bam*HI and then cloned into plasmid pJTU1278 (also digested with *Spe*I and *Bam*HI) to obtain the recombinant plasmid pJTU-cagRS.

Deletion of *cagRS* was performed by double recombination between the *S. clavuligerus* F613-1 genome and the pJTU1278-derived plasmid pJTU-cagRS, resulting in the knockout of the *cagRS* genes (Figure 1). Conjugation was performed using *S. clavuligerus* F613-1 and *E. coli* ET12567/pUZ8002 as described (Sambrook et al., 1989; Kieser et al., 2000). The Δ*cagRS* mutant strain was confirmed by PCR.

For complementation, *cagRS* was amplified with the primers CagRS com-F and CagRS com-R, generating a fragment carrying the coding sequence and the *cagRS* promoter, and cloned into *Nde*I/*Xba*I-cut pSET152 to create pSET-cagRS. The plasmids pSET152 and pSET-cagRS were introduced individually into the Δ*cagRS* mutant by conjugation.

### Construction of a CagR-3 × Flag-Complemented *S. clavuligerus* Strain

To engineer an *S. clavuligerus* strain expressing CagR with a C-terminal, triple-Flag tag (DYKDHDGDYKDHDIDYKDDDDK), the pSET152-derived construct pSET-cagRFlag was created via assembly of multiple DNA fragments using the Gibson Assembly Cloning Kit (New England BioLabs). To avoid folding of the 3 × Flag tag into the inside of the CagR protein, the [Gly_4_Ser]_3_ linker (GGGGSGGGGSGGGGS) (Bush et al., 2013) was inserted between the 3 × Flag tag and the coding region of *cagR*. The flow chart for construction of the recombinant plasmid pSET-cagRFlag is shown in Supplementary Figure S1. The recombinant plasmid pSET-cagRFlag was confirmed by DNA sequencing, and then pSET-cagRFlag was introduced into the Δ*cagRS* mutant by conjugation, and its ability to restore CA production was assessed during liquid-state fermentation.

### Bioassay and HPLC Analysis of CA Production

The concentration of CA was analyzed by bioassay analysis and HPLC analysis. The indicator *E. coli* strain MA18 was spread on LB solid medium supplemented with 100 μg/mL ampicillin (Jones et al., 2005). Briefly, for the bioassay analysis, *S. clavuligerus* strains were grown on BSCA plates for 3–9 days, on MSF solid media for 5–11 days, on MM solid media for 9–15 days, or on ISP4 solid media for 7–13 days, and then the corresponding agar blocks were excavated with a 6 mm punch and transferred onto the LB agar plates. The diameter of the inhibition zone was gauged after overnight culturing at 37°C. The concentration of CA during the liquid-state fermentation was detected by HPLC with an Inertsil ODS-3 4.6 mm × 150 mm, 5 μm column (Jin et al., 2015; Qin et al., 2017), using clavulanate lithium (provided by Lunan Pharmaceutical Co.) as the standard for quantification. For bioassay and HPLC analysis of CA production, the experiments were conducted in triplicate. Statistical analysis was performed using SPSS Statistics V19.0 software.

### Transcriptome Sequencing and Analysis

For transcriptome sequencing and analysis, mycelium of *S. clavuligerus* F613-1 and its derivative Δ*cagRS* were harvested from fermentation liquid at 72 h and rapidly frozen in liquid nitrogen. Total RNA of F613-1 and Δ*cagRS* was purified using an RNA extraction kit (SBSBIO, Beijing China) according to the manufacturer’s protocol. The extracted total RNA samples were then treated with RNase-free DNase I (Invitrogen) twice according to the recommended protocols to remove the residual chromosomal DNA. Two sets of RNA for both strains were prepared separately. The integrity of total RNA was determined using a NanoDrop One C (Thermo Fisher Scientific), and the RNA Integrity Number value of each sample met the standard required for preparing a cDNA library. The cDNA libraries were prepared according to the manufacturer’s instructions (Illumina). Briefly, the ribosomal RNA in 1–4 μg total RNA was removed using Ribo-zero rRNA Removal solution (Illumina), leaving only mRNA. The mRNA was then fragmented, to an average fragment length of about 200 nt, and reverse transcribed into single-stranded cDNA using random hexamer priming. When the second cDNA strand was synthesized, the dTTP was replaced by dUTP. The cDNA fragments with a single ‘A’ base overhang at their 3′-ends were obtained after end-repair and 3′-adenylation. Adapters were then ligated to the ends of the cDNA fragments. Fifteen rounds of PCR amplification were performed to enrich the adapter-modified cDNA library using primers complementary to the ends of the adapters, and PCR products were purified using Ampure XP beads (Agencourt). The ready-to-sequence Illumina library was quantified as previously described (Zhang et al., 2017).

The transcriptome was sequenced using a HiSeq 3000 sequencer (Illumina) at RibBio Corporation (Shenzhen, China). A paired-end, 2 × 150 bp sequencing strategy was used, and more than 1 Gb data was obtained for each sample. The sequencing generated 6 files of reads corresponding to F613-1 and Δ*cagRS*, with three replicates for each. After filtering the raw data, removing the linker sequences, low-quality reads and the residual rRNA sequences, the remaining data were marked as the effective reads. The effective reads could be obtained using fastx_clean^1^, a home-made software package based on the FASTX toolkit^2^ and SortMeRNA (excludes the ribosomal RNA-like reads) (Kopylova et al., 2012). The effective reads were used for subsequent genome comparisons, and the distribution of reads compared to the genome was statistically analyzed. For Δ*cagRS*, 93.58% of effective reads were distributed in ORFs (open reading frames), and 6.42% in intergenic regions. For F613-1, 95.18% of effective reads were distributed in exonic regions, and 4.82% in intergenic regions. The expression level of each gene was normalized by the number of reads per kilobase of transcriptome per million mapped reads (RPKM). The differentially expressed genes were selected using the Audics program with parameters of |log2FoldChange| > 1 and *q*-value < 0.001. The transcriptome data has been deposited in the Gene Expression Omnibus database^3^. The accession number is GSE119208.

### Real-Time Quantitative PCR Analysis

Total RNA isolation and real-time quantitative PCR (RT-qPCR) procedures were performed as described previously (Fu et al., 2017). Mycelium of *S. clavuligerus* F613-1 and Δ*cagRS* were harvested from fermentation liquid at 24, 72, 120, 168, and 216 h, rapidly frozen in liquid nitrogen and then total RNA was extracted using an RNA extraction kit (SBSBIO, Beijing China) according to the manufacturer’s protocol. Total RNA samples were treated with Turbo DNA-free reagents (Ambion, United States) to remove the residual chromosomal DNA. The cDNAs were synthesized using random hexamer primers (pdN6, Amersham Pharmacia Biotech, England), M-MLV reverse transcriptase (Invitrogen, England) and dNTPs (Roche, Switzerland). Real-time PCR assays were performed on the Roche LightCycler 480 using SYBR Green Mix (ToYoBO, Osaka, Japan). Relative quantities of cDNA were normalized to the amounts of 16S rRNA. For RT-qPCR assays, experiments were conducted in triplicate. Statistical analysis was performed using SPSS Statistics V19.0 software.

### Chromatin Immunoprecipitation and DNA Sequencing Assay (ChIP-Seq Assay)

Chromatin immunoprecipitation assay was performed as previously described (Bush et al., 2013). *S. clavuligerus* CagR-Flag (Δ*cagRS*:: *cagR*-[Gly_4_Ser]_3_-3^∗^Flag-*cagS*) was grown in four 50-mL SCF at 25°C for 72 h. Formaldehyde was added to cultures at a final concentration of 1% (vol/vol) for 30 min. Glycine was then added to a final concentration of 125 mM to stop the cross-linking. The samples were left at room temperature for 5 min and washed twice in PBS (phosphate-buffered saline, pH 7.4) buffer. The pellets were resuspended in 5 mL of ChIP-lysis buffer [10 mM Tris-HCl, pH 8.0, 50 mM NaCl, 1 mM phenylmethanesulfonyl fluoride (PMSF) and 15 mg/mL lysozyme] and incubated at 37°C for 30 min or until lysed. Then, 5 mL ChIP-IP buffer (50 mM Tris-Cl, pH 8.0, 250 mM NaCl, 0.8% [vol/vol] Triton X-100 and 1 mM PMSF) was added, and the samples were chilled on ice. Sonication was performed at a high-power setting for 40 cycles (10 s on and 10 s off) using an ultrasonic processor (FS-250N, Shengxi Ultrasonic Instrument Co., Ltd., Shanghai) to shear chromosomal DNA into fragments ranging from 200 to 500 bp on average. The samples were centrifuged at 12,000 rpm at 4°C for 10 min to collect the supernatant, after which 50 μL of each was set aside for total DNA extraction (input). Next, 40 μL of IgG-agarose (Sigma-Aldrich, A0919) was washed according to the manufacturer’s instructions and added to the remaining lysates. The mixtures were then incubated on a rotating wheel at 4°C for 2 h, and then the samples were centrifuged at 5,500 g at 4°C for 30 s to collect the supernatants. Next, the 40 μL anti-FLAG M2 Affinity Gel (Sigma-Aldrich, A2220) was washed according to the manufacturer’s instructions and added to the above supernatants. The mixtures were then incubated on a rotating wheel at 4°C overnight. The samples were centrifuged at 5,500 g at 4°C for 30 s, and the pellets were washed twice with 0.5 × ChIP-IP buffer and then twice with 1 × ChIP-IP buffer and transferred to new tubes after the first washing step. The pellets and 50 μL of total cell extracts (set aside earlier) were eluted overnight at 65°C in 100 μL of ChIP-IP elution buffer (50 mM Tris-HCl, pH 7.6, 10 mM EDTA and 1% SDS) to reverse the cross-links. The samples were centrifuged at 12,000 rpm for 5 min to remove the beads. The pellets were extracted with 50 μL of TE buffer (10 mM Tris-Cl, pH 7.4 and 1 mM EDTA) and incubated with 0.2 mg/mL proteinase K (Sigma) and 20 μg/mL RNase A (Sigma) for 2 h at 55°C. The samples were extracted twice with phenol-chloroform and once with chloroform and further purified using Bioteke columns (Bioteke, Beijing). DNA was quantified using a Nano-Drop spectrophotometer (Thermo Scientific).

ChIP-seq libraries were prepared and sequenced on a HiSeq 2500 sequencer (Illumina, Novogene) by Novogene Science and Technology Co., Ltd. BigWig files were generated from the alignment for visualization purposes (Kent et al., 2010). MACS2 (Model-based Analysis of ChIP-seq) was used to identify peaks using a *p*-value ≤ 0.005 (Zhang et al., 2008). To identify possible binding motifs of the CagR DNA-binding response regulator, the ChIP peak sequences were analyzed by Dreme software (Bailey and Elkan, 1994; Bailey, 2011).

### Overexpression and Purification of His-Tagged CagR

The *cagR* gene of *S. clavuligerus* F613-1 was amplified using primer pairs cagRHis-F/R (Supplementary Table S2) and then inserted into the pMD18T vector to generate the intermediate recombinant plasmid pMD18T-cagR. After confirmation by DNA sequencing, *cagR* from pMD18T-cagR was cloned into the pET-15b expression vector, producing the recombinant plasmid pET-cagR. Finally, pET-cagR was introduced into *E. coli* BL21(DE3) for protein expression. His-tagged CagR protein was induced and purified as previously described (Fu et al., 2017). The purity of His-tagged CagR protein was determined on a 10% SDS-PAGE gel.

### Electrophoretic Mobility Shift Assays (EMSAs)

PCR was used to amplify 150–300 bp fragments from the intergenic regions of genes using genomic DNA of F613-1 as template (primers are listed in Supplementary Table S2). The amplified DNA fragments were labeled at the 3′-end with biotin-11-UTP using the Biotin 3′ End DNA Labeling kit (Thermo Scientific), according to the manufacturer’s instruction. Non-specific cold probes (PolydI/dC) were added to control reaction mixtures as competitors. EMSAs were carried out as described previously (Zhang et al., 2015).

## Results

### Deletion of *cagRS* Results in Decreased Production of CA

*Streptomyces clavuligerus* F613-1 is an industrial CA producer, and its complete genome sequence has been reported (Cao et al., 2016). The putative TCS CagRS is located near the CA biosynthetic gene cluster in F613-1 (Figure 1C). To characterize the function of this TCS, a *cagRS* null mutant of F613-1 was constructed, and a complemented strain, Δ*cagRS*com, was also constructed by expressing *cagRS in trans* in the φC31 integration site in Δ*cagRS* (Figure 1A). Deletion and complementation were confirmed by PCR (Figure 1B).

**FIGURE 2:**
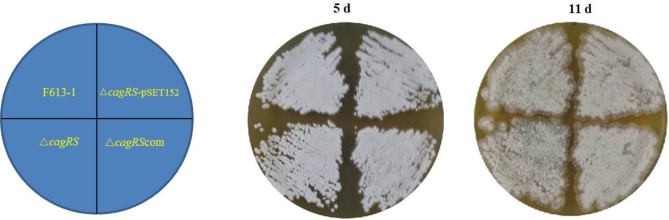
Phenotype of F613-1, Δ*cagRS*, Δ*cagRS*-pSET152, and Δ*cagRS* com on BCSA solid medium.

On different media (BSCA, MSF, MM, and ISP4), no growth or phenotypic differences were noted for Δ*cagRS* compared with the parental strain F613-1 (Figure 2). Interestingly, a bioassay revealed that deletion of *cagRS* resulted in a modest reduction in CA biosynthesis, as indicated by the smaller diameter of the inhibition zone produced by Δ*cagRS* compared to that of the wild-type strain F613-1 (data not shown).

**FIGURE 3:**
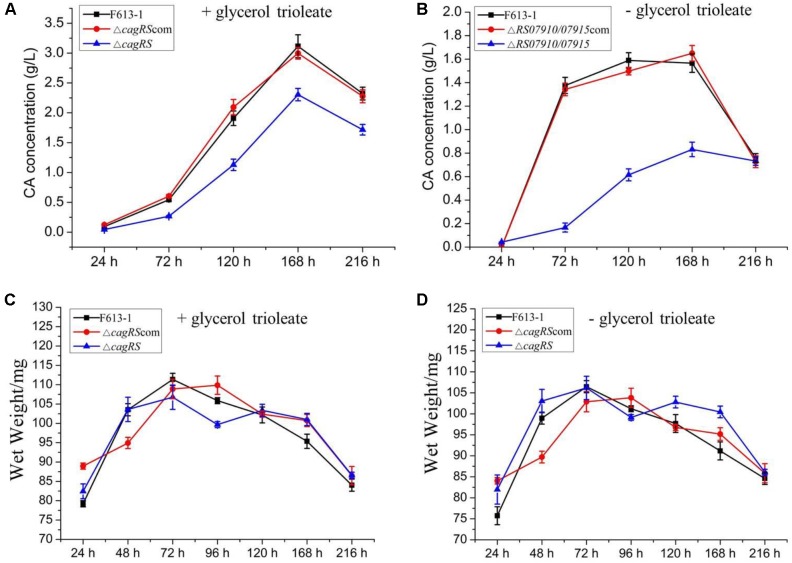
Clavulanic acid (CA) liquid fermentation titers of F613-1, Δ*cagRS* and the complemented strain. Analysis of the change of CA concentration during fermentation **(A,B)**. For detecting CA production during the liquid fermentation process, spores (10^6^/mL) were inoculated into 100 mL SCZ seed medium and cultured at 25°C, 200 rpm for 48 h to obtain seed cultures. Next, 5 mL seed cultures were transferred to 100 mL SCF fermentation medium supplemented with **(A)** or without **(B)** 1.6% (v/v) glycerol trioleate, and then grown at 25°C, 200 rpm for 216 h. To analyze CA concentration, 1 mL samples of fermentation liquid were collected at 24, 72, 120, 168, and 216 h, and centrifuged at 12000 rpm, 4°C, 5 min to collect the supernatant. Next, HPLC was performed immediately after the supernatant was filtered with a 0.22 mm filter membrane. **(C,D)** Growth curves of F613-1, Δ*cagRS* and Δ*cagRS*com with **(C)** or without **(D)** glycerol trioleate. Samples for growth curve analysis were harvested at seven time points (24, 48, 72, 96, 120, 168, and 216 h). Data are the mean ± SD of three separate experiments.

To examine the effect of CagRS on CA production in more detail, an HPLC assay was performed to quantitatively analyze the production of CA in Δ*cagRS* and F613-1. No differences were detected in the biomasses of Δ*cagRS* and F613-1 (Figure 3C,D). However, the CA concentration produced by Δ*cagRS* in fermentation medium supplemented with glycerol trioleate was decreased by 50.8% (*t* = 41, 63, *P* < 0.05) at 72 h, 40.9% (*t* = 69, 103, *P* < 0.05) at 120 h, 26.0% (*t* = 122, 190, *P* < 0.05) at 168 h, and by 26.1% (*t* = 109, 125, *P* < 0.05) at 216 h, when compared with production by F613-1 (Figure 3A). It was reported that the addition of glycerol trioleate could enhance CA production in *S. clavuligerus* NRRL 3585 (Kim et al., 2009). In this study, when compared with F613-1 levels, the CA concentrations produced by Δ*cagRS* in fermentation medium without glycerol trioleate were decreased by 87.9% (*t* = 32, 75, *P* < 0.05) at 72 h, 61.3% (*t* = 50, 101, *P* < 0.05) at 120 h, and 46.9% (*t* = 58, 99, *P* < 0.05) at 168 h (Figure 3B). The results revealed that CA production in Δ*cagRS* is decreased when compared with F613-1 whether or not glycerol trioleate is present in the fermentation medium, consistent with the bioassay results. Additionally, CA production by Δ*cagRS* dropped more markedly in the absence of glycerol trioleate supplementation, indicating that *cagRS* may affect metabolic processing of the CA direct precursor glyceraldehyde 3-phosphate (G3P).

Δ*cagRS* was almost fully complemented by a wild-type copy of *cagRS* introduced into the φC31 integration site (Figure 2, 3), suggesting that the TCS CagRS contributes to regulation of CA production, although it does not affect the phenotype of *S. clavuligerus* F613-1.

### Transcriptome Analysis Revealed That *cagRS* Regulates CA Production via Multiple Pathways

To gain further insights into the mechanisms by which CagRS regulates CA biosynthesis, transcriptome analysis was performed. Transcriptome sequencing revealed that 5943 genes were expressed in F613-1 under the test conditions, and 5843 genes were expressed in Δ*cagRS*. Statistical analysis showed that 2036 genes showed significant differences in expression (log2 FC > 1.0) between Δ*cagRS* and F613-1 during fermentation, with 1286 of these genes upregulated in Δ*cagRS*. The differentially expressed genes were further analyzed by Gene Ontology (GO) term enrichment analysis^4^ and KEGG pathway analysis^5^. Significant enrichment in GO terms was found mainly in the categories of “metabolic process,” “oxidation-reduction process,” and “regulation of transcription” (Supplementary Figure S3A). KEGG pathway analysis revealed that many of the differentially expressed genes are involved in fatty acid biosynthesis, pyruvate metabolism, arginine biosynthesis and carbon metabolism (Supplementary Figure S3B). RT-qPCR was also performed to validate the transcriptome results, and the results for the tested genes were in general agreement with the transcriptome results except for *oat2* (Table 1).

**Table 1:** Differential expression of genes associated with G3P and arginine metabolism and CA biosynthesis in Δ*cagRS*.

Gene ID in ATCC 27064	Gene ID in F613-1	Fold change transcriptome^c^	Fold change RT-qPCR^d^	Product	Pathway	*p*-value
**Genes involved in G3P and arginine metabolism**						
*SCLAV_1867*^a^ (*gpmA1)*	*BB341_RS18990*^b^	0.17	0.39	Phosphoglycerate mutase	Glycolysis	0
*SCLAV_1059*(*fucA)*	*BB341_RS22720*	2.00	3.73	Class II aldolase family protein	Glycolysis	6.66E-16
*SCLAV_2648*	*BB341_RS15270*	2.06	4.12	Class II fructose-bisphosphate aldolase	Glycolysis	1.11E-15
*SCLAV_0289*	*BB341_RS26335*	0.49	0.33	ROK family protein	Glycolysis	1.30E-39
*SCLAV_3958*	*BB341_RS21415*	0.46	0.24	Glucokinase	Glycolysis	1.66E-70
*SCLAV_1613(aceE)*	*BB341_RS13605*	0.33	0.45	Pyruvate dehydrogenase E1	Glycolysis	0
*SCLAV_4928(poxB)*	*BB341_RS21120*	0.46	0.53	Pyruvate dehydrogenase	Glycolysis	0
*SCLAV_5509*	*BB341_RS01000 (gap2)*	0.29	0.37	Glyceraldehyde-3-phosphate dehydrogenase	Glycolysis	0
*SCLAV_0879*	*BB341_RS23575*	0.29	0.31	Glycerol-3-phosphate dehydrogenase/oxidase	Glycerol metabolism	9.96E-184
*SCLAV_0228*	*BB341_RS26625*	51.98	9.94	Acyl-CoA dehydrogenase	Fatty acid degradation	6.66E-16
*SCLAV_2974(paaH)*	*BB341_RS03665(paaH)*	2.99	2.08	3-hydroxybutyryl-CoA dehydrogenase	Fatty acid degradation	6.66E-16
*SCLAV_4820*	*BB341_RS04820*	11.89	8.69	Acyl-CoA dehydrogenase	Fatty acid degradation	4.44E-16
*SCLAV_4816(echA5)*	*BB341_RS04840*	3.10	2.16	Enoyl-CoA hydratase	Fatty acid degradation	2.22E-16
*SCLAV_0801(argC)*	*BB341_RS23945 (argC)*	9.61	2.12	*N*-acetyl-gamma-glutamyl-phosphate reductase	Arginine synthesis	1.11E-15
*SCLAV_0800(argJ)*	*BB341_RS23950 (argJ)*	7.33	6.45	Bifunctional ornithine	Arginine synthesis	1.11E-15
*SCLAV_0799(argB)*	*BB341_RS23955 (argB)*	6.74	5.48	Acetylglutamate kinase	Arginine synthesis	1.11E-15
*SCLAV_0798(argD)*	*BB341_RS23960 (argD)*	5.33	5.67	Acetylornithine aminotransferase	Arginine synthesis	1.11E-15
*SCLAV_0797*	*BB341_RS23965 (argR)*	5.04	5.49	Arginine repressor	Arginine synthesis	1.11E-15
*SCLAV_0796*	*BB341_RS23970 (argG)*	3.47	2.63	Argininosuccinate synthase	Arginine synthesis	1.11E-15
*SCLAV_0795*	*BB341_RS23975 (argH)*	4.70	3.47	Argininosuccinate lyase	Arginine synthesis	1.11E-15
**Genes involved in CA biosynthesis**						
*SCLAV_4185*	*BB341_RS07870 (orf14)*	0.40	0.34	GNAT family acetyltransferase	Biosynthesis of CA	0
*SCLAV_4190*	*BB341_RS07845 (car)*	0.35	0.48	Oxidoreductase	Biosynthesis of CA	0
*SCLAV_4193(oat2)*	*BB341_RS07830 (oat2)*	2.81	0.89	Ornithine acetyltransferase	Biosynthesis of CA	1.11E-15
*SCLAV_4197*	*BB341_RS07810 (ceaS2)*	0.40	0.7	*N*(2)-(2-carboxyethyl)arginine synthase	Biosynthesis of CA	0
*SCLAV_0471(avaA2)*	*BB341_RS25520(avaA2)*	2.60	5.57	Gamma-butyrolactone biosynthesis protein	Regulation of CA	2.17E-13


*^*a*^locus_tag in Streptomyces clavuligerus ATCC 27064. ^*b*^locus_tag in Streptomyces clavuligerus F613-1. ^*c*^Results show fold change in ΔcagRS compared with F613-1 as determined by transcriptome analysis. ^*d*^******Results show fold change ΔcagRS compared with F613-1 as determined by RT-qPCR. Red lettering indicates down-regulated genes. Green lettering indicates up-regulated genes*.

Arginine and G3P are two direct precursors of CA (Townsend and Meng-Fei, 1985; Khaleeli et al., 1999), and therefore, arginine and G3P metabolism may affect CA production. As shown in Table 1, the expression levels of all of the genes in the arginine biosynthetic gene cluster increased significantly in Δ*cagRS* compared with levels in F613-1, and the expression levels of many genes involved in G3P metabolism also changed notably in Δ*cagRS* compared with F613-1, suggesting that CagRS regulates arginine and G3P metabolism. In addition, the expression levels of *ceaS2, oat2, car*, and *orf14*, genes related to CA biosynthesis, were significantly changed in Δ*cagRS* when compared with F613-1 levels, suggesting that CagRS regulates the CA biosynthetic gene cluster. The above transcriptome data indicate that the TCS CagRS may affect CA production both directly, through the CA biosynthetic gene cluster, and indirectly, by affecting arginine and G3P metabolism.

### ChIP-Seq Analysis of the *in vivo* Targets of the Response Regulator CagR

In order to further determine which genes are directly regulated *in vivo* by CagRS in *S. clavuligerus* F613-1, ChIP-seq analysis was conducted. Strain CagR-Flag was first constructed, which lacks CagRS at its native locus but expresses CagR and a C-terminal, triple Flag-tagged version of CagR. No growth or phenotypic differences were noted for strain CagR-Flag when compared with F613-1 and Δ*cagRS*, and the CA concentration of strain CagR -Flag was similar to F613-1 levels (Supplementary Figure S2A). Furthermore, CagR-[Gly4Ser]-3 × Flag was readily detected using anti-Flag antibody in western blot assays, without visible cross-reaction with any other protein, and the highest peak of CagR-[Gly4Ser]-3 × Flag expression emerged at 72 h during fermentation (Supplementary Figure S2B), suggesting optimal conditions for clean ChIP-seq experiments. Therefore, the mycelium of strain CagR-Flag was collected at 72 h during fermentation, and the ChIP-seq assay was conducted with Anti-FLAG M2 affinity gel. In addition, the total (non-immunoprecipitated) input DNA was used as a negative control.

**FIGURE 4:**
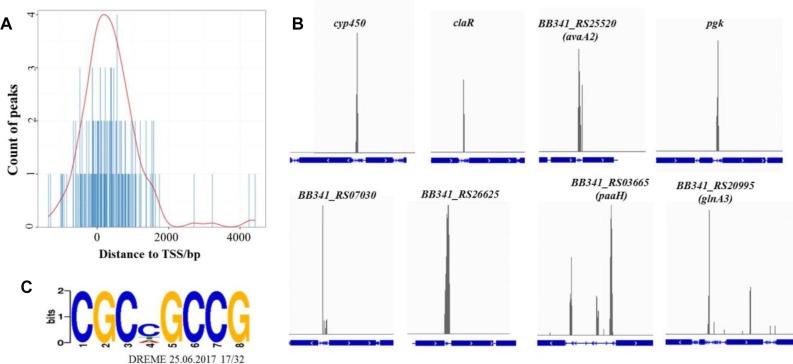
ChIP-seq data for the CagR target genes. **(A)** The distance between peak and transcription start site. TSS, transcription start site. The TSS of each peak-related gene is detected by Peak Annotator (Salmon-Divon et al., 2010). The count of peaks is calculated according to the distance between the peak and the TSS. **(B)** ChIP-seq peaks for eight selected CagR target genes: *cyp450, claR, avaA2, pgk, BB341_RS07030, BB341_RS26625, paaH*and *glnA3*. The blue boxes represent the gene coding regions, and the blue lines represent the intergenic regions. The eight corresponding ChIP-seq peak values for the negative control (input) dropped dramatically (data not shown). **(C)** The consensus binding sequence for CagR was determined by DREME (Discriminative Regular Expression Motif Elicitation) (Bailey, 2011). The height of the letters in the sequence logo, in bits, is proportional to the frequency of the A, C, T, or G nucleotides at each position of the motif. This sequence motif in *S. clavuligerus* F613-1 is nearly identical for that in *S. clavuligerus* ATCC27064.

The average DNA fragment sizes for the input and anti-Flag ChIP libraries were 294 and 253 bp, respectively. The input library had 12.74 million reads, and the Flag antibody ChIP library had 17.26 million reads. Over 95% of the reads were mapped to the *S. clavuligerus* F613-1 genome. The locations of the enriched peaks identified by the MACS2 program in the *S. clavuligerus* F613-1 genome are presented in a Supplemental Table (Additional File S2A). All 162 enriched regions were mapped to previously annotated genes in *S. clavuligerus* F613-1. Of those 162 CagR ChIP-seq targets, 22 targets are transcriptional regulators, and 41 targets are hypothetical proteins. In addition, eight targets of CagR are potentially involved in CA biosynthesis (Table 2 and Figure 4B): two genes (*cyp450* and *claR*) located in the CA biosynthetic gene cluster; the *BB341_RS25520* (*avaA2*) gene, as it encodes a gamma-butyrolactone biosynthesis protein and the γ-butyrolactone signaling system affects CA biosynthesis (Santamarta et al., 2005); *pgk*, encoding phosphoglycerate kinase; *BB341_RS07030, BB341_RS26625* and *BB341_RS03665* (*paaH*), which are involved in fatty acid degradation; and *BB341_RS20995* (*glnA3*), a gene encoding glutamine synthetase, which is involved in arginine biosynthesis. Moreover, transcriptome analysis revealed that the expression level of the *BB341_RS25520* (*avaA2*), *BB341_RS26625* and *BB341_RS03665* (*paaH*) genes increased significantly in Δ*cagRS* compared with F613-1 (Table 1).

**Table 2:** Clavulanic acid (CA) biosynthesis-associated genes identified as CagR targets by ChIP-seq.

Gene ID in ATCC 27064	Gene ID in F613-1	Biological function	Pathway	-Log10 (*p*-value)
*SCLAV_4189(cyp450)*	*BB341_RS07850 (cyp450)*	Cytochrome P450	Biosynthesis of CA	3.05595
*SCLAV_4191(claR)*	*BB341_RS07840 (claR)*	Transcriptional regulator	CA biosynthesis regulation	4.0205
*SCLAV_0471(avaA2)*	*BB341_RS25520(avaA2)*	Gamma-butyrolactone biosynthesis protein	CA biosynthesis regulation	2.53812
*SCLAV_1147 (pgk)*	*BB341_RS22330 (pgk)*	Phosphoglycerate kinase	Glycolysis	3.0029
*SCLAV_4367*	*BB341_RS07030*	Enoyl-CoA hydratase	Fatty acid degradation	2.33484
*SCLAV_0228*	*BB341_RS26625*	Acyl-CoA dehydrogenase	Fatty acid degradation	3.83473
*SCLAV_2974(paaH)*	*BB341_RS03665(paaH)*	3-hydroxybutyryl-CoA dehydrogenase	Fatty acid degradation	4.78544
*SCLAV_1431(glnA3)*	*BB341_RS20995(glnA3)*	Glutamine synthetase	Arginine synthesis	2.80523


*The genes listed in this table are limited to those associated with CA biosynthesis*.

Statistical analysis showed that the 162 enriched peaks were concentrated near transcriptional start sites, suggesting that CagR is a transcriptional regulatory protein (Figure 4A). Using the motif discovery algorithm DREME (Discriminative Regular Expression Motif Elicitation) (Bailey, 2011), the most significantly enriched motif within the peak regions was CGCNGCCG (*P*-value of 3.3e-10 and an *E*-value of 3.9e-009) (Figure 4C). Approximately 78% of the potential CagR targets had a CGCNGCCG motif correlating closely with the position of the ChIP-seq peak. Bioinformatics analysis found that the CGCNGCCG motif is also present in almost all homologous genes, i.e., the potential CagR target genes, in *S. clavuligerus* ATCC 27064. Of the 162 CagR ChIP-seq targets, approximately 35% of the associated genes showed a greater than twofold change in expression when comparing Δ*cagRS* to F613-1 in transcriptome analysis (Additional File S2B). Strikingly, 21% of the genes identified by ChIP-seq as CagR targets were upregulated significantly, and 16% were downregulated significantly, in Δ*cagRS*. These findings suggest that CagR is bifunctional, working almost equally as an activator and as a repressor to control differentiation in *S. clavuligerus.*

### *cagRS* Regulates the Biosynthesis of Arginine, a Direct Precursor of CA

**FIGURE 5:**
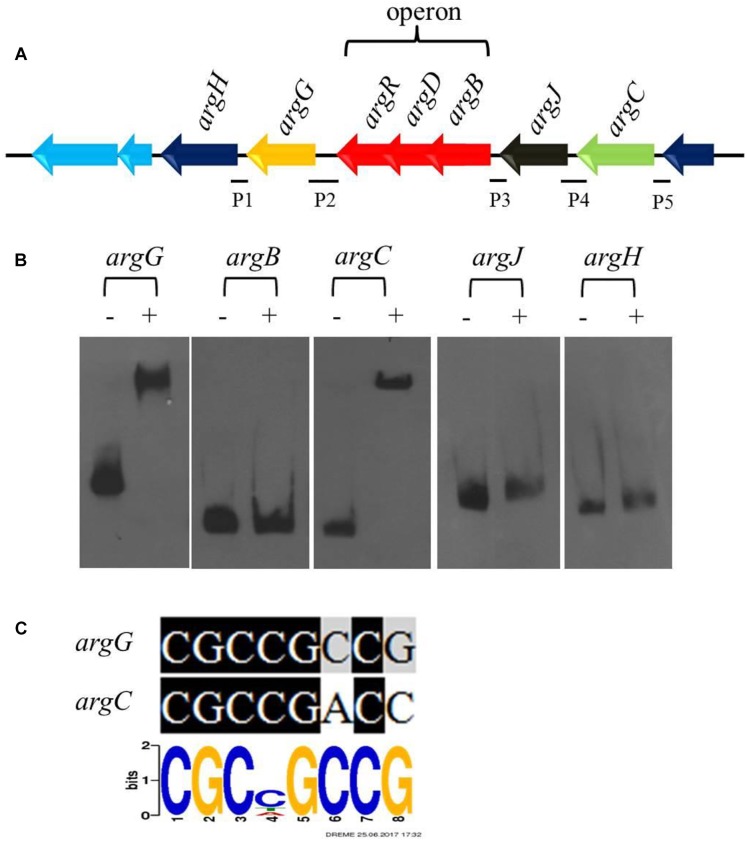
CagRS regulates the biosynthesis of arginine-the direct precursor of CA. **(A)** Schematic diagram of the arginine biosynthetic gene cluster. P1–P5 are different promoter regions of the arginine gene cluster: P1, 211 bp of the *argH* upstream region; P2, 299 bp of the *argG* upstream region; P3, 154 bp of the *argB*-*D*-*R* upstream region; P4, 271 bp of the *argJ* upstream region; P5, 160 bp of the *argC* upstream region. **(B)** The binding of CagR to the promoter regions of *argC* and *argG.* EMSAs of *argG, argB, argC, argJ*, and *argH* with purified His-tagged CagR. The promoter fragments were labeled with biotin-11-UTP using the Biotin 3′ End DNA Labeling kit. The above probes were incubated either with no protein (–) or 3.0 μg CagR (+). The appropriate amount of polydI/dC (1.0 mg) was used as competitor. **(C)** Comparison of *argG* and *argC* promoter sequences with the conserved CGCNGCCG motif for CagR binding.

Arginine is one of the direct precursors for CA biosynthesis (Liras et al., 2008). Seven genes (*argH, argG, argR, argD, argB, argJ*, and *argC*) involved in the biosynthesis of arginine are located in the arginine biosynthetic gene cluster, in which *argR, argD*, and *argB* form an operon (Figure 5A). Transcriptome and RT-qPCR analyses revealed that the expression of the gene cluster increased significantly in Δ*cagRS* compared with F613-1 (Table 1). As deletion of CagRS was associated with activation of these seven consecutive genes, we speculated that CagR may interact directly with one or more of the intergenic regions in the arginine gene cluster. Therefore, we amplified five intergenic regions, i.e., the intergenic regions of *argB, argC, argJ, argH*, and *argG*, to use as probes in EMSAs. Obvious shifting was only observed with the intergenic region probes for *argG* and *argC* (Figure 5B and Supplementary Figure S5), and notably, these two intergenic regions contain sequences identical (*argG*) or highly similar (*argC*) to the conserved CGCNGCCG motif sequence (Figure 5C), consistent with the binding of CagR to these intergenic regions. However, we did not detect interactions between CagR and the intergenic region probes for the *argB*-*D*-*R* operon, *argJ* or *argH*, although the transcription levels of these genes also increased significantly in the Δ*cagRS* strain.

### *cagRS* May Regulate G3P Metabolic Processes

G3P is another direct primary metabolic precursor of CA (Khaleeli et al., 1999; Liras et al., 2008), and glyceraldehyde-3-phosphate dehydrogenases (GAPDHs) are responsible for catalyzing the formation of G3P into 1,3-diphosphoglycerate. Two genes (*gap1* and *gap2*) encoding distinct GAPDHs have been characterized in *S. clavuligerus* 27064 (Li and Townsend, 2006). In this study, the expression level of *gap2* in Δ*cagRS* was reduced significantly compared to levels in F613-1 (Table 1), whereas *gap1* showed no significant differences in expression between the two strains. *BB341_RS23575*, which encodes glycerol-3-phosphate dehydrogenase, an enzyme involved in converting glycerol into G3P, was also significantly down-regulated in the mutant.

In addition, transcriptome analysis and RT-qPCR revealed that many genes involved in glycolysis, such as *BB341_RS18990, BB341_RS26335, BB341_RS21415, BB341_RS13605*, and *BB341_RS21120*, were also significantly down-regulated in Δ*cagRS* compared with F613-1 (Table 1). Furthermore, ChIP-seq assays revealed that *BB341_RS22330* (*pgk*), encoding the phosphoglycerate kinase involved in glycolysis, may be directly regulated by CagR *in vivo* (Table 2 and Figure 4B). Both glycolysis and the glycerol-converting process affect G3P concentration, as summarized in Figure 8A, and our transcriptional data revealed that G3P concentration may be decreased in Δ*cagRS* when compared with F613-1.

Glycerol trioleate, which dissociates into glycerol and oleic acid, was reported to enhance CA production in *S. clavuligerus* (Kim et al., 2009), with the produced glycerol converted into G3P for CA production. We found that, although CA production decreased significantly in the absence of glycerol trioleate, CA concentration increased from 72 h to 168 h continuously in Δ*cagRS*, indicating that *cagRS* may affect primary metabolic processing of the direct CA precursor G3P.

### *cagRS* Modulates Expression of the CA Biosynthetic Gene Cluster

**FIGURE 6:**
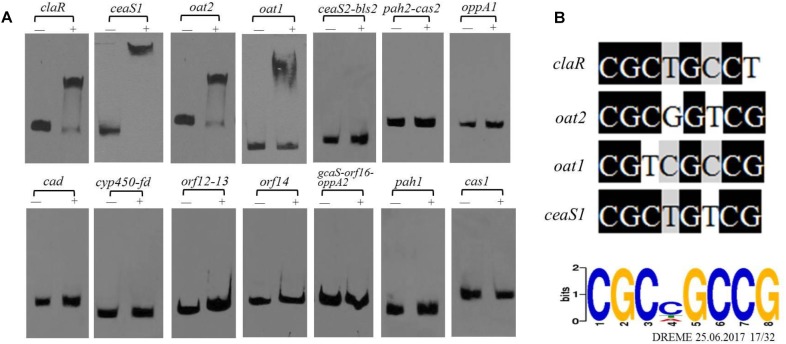
The binding of CagR to the promoter region of CA cluster genes. **(A)** The upstream promoter region of CA cluster genes are, respectively: 181 bp of the *claR* upstream region; 214 bp of the *ceaS1* upstream region; 169 bp of the *oat2* upstream region; 171 bp of the *oat1* upstream region; 234 bp of the *ceaS2*-*bls2* upstream region; 196 bp of the *pah2*-*cas2* upstream region; 158 bp of the *oppA1* upstream region; 191 bp of the *car* upstream region; 157 bp of the *cyp450*-*fd* upstream region; 246 bp of the *orf12*-*orf13* upstream region; 137 bp of the *orf14* upstream region; 171 bp of the *gcaS*-*orf16*-*oppA2* upstream region; 213 bp of the *pah1* upstream region; 183 bp of the *cas1* upstream region. The probes were incubated either with no protein (–) or 3.0 μg CagR (+). The appropriate amount of polydI/dC (1.0 mg) was used as competitor. **(B)** Comparison of *claR, oat2, oat1* and *ceaS1* promoter sequences with the conserved motif for CagR binding.

As noted previously, gene clusters related to CA biosynthesis include the CA biosynthetic gene cluster, the paralog gene cluster and the clavam gene cluster (Figure 1C). Both the transcriptome and ChIP-seq analyses indicated that CagRS might affect the genes involved in CA biosynthesis (Tables 1, 2), so we speculated that CagR may interact directly with one or more of the promoters in the CA biosynthesis-related gene clusters. To investigate the targets of CagR, we amplified 14 promoter fragments (Figure 6A), which covered the upstream regions for 20 transcripts localizing to these gene clusters, to use as probes in EMSAs. Only the *cas1* gene in the clavam gene cluster is involved in CA biosynthesis, but when incubated with purified His-tagged CagR, EMSA assays showed that CagR does not bind the intergenic region of *cas1* (Figure 6A). *ceaS1, bls1, pah1*, and *oat1* in the paralog gene cluster are also involved in CA biosynthesis, and EMSA assays showed that CagR does not bind the intergenic region of the *bls1*and *pah1* genes, but CagR could bind the intergenic region of the *ceaS1* and *oat1* genes (Figure 6A and Supplementary Figure S5). For genes in the CA biosynthetic gene cluster, EMSA assays showed that CagR could bind the intergenic region of *claR* and *oat2* genes (Figure 6A and Supplementary Figure S5). DNA sequence alignment also showed that promoter regions of *claR, ceaS1, oat1*, and *oat2* contain sequences with strong similarity to the conserved CGCNGCCG motif (Figure 6B) in F613-1.

**FIGURE 7:**
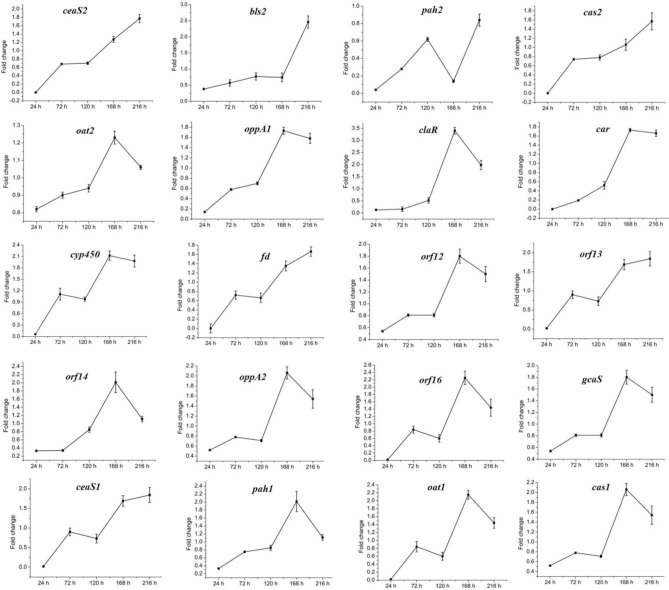
Expression of CA cluster genes by RT-qPCR in F613-1 and Δ*cagRS*. Results were normalized for *16S rDNA* gene content and are shown as fold change over the F613-1 control, which was given a value of 1. Fold change: expression level in Δ*cagRS* compared with F613-1. Data are the mean ± SD of three separate experiments.

The above data indicated that CagRS is directly associated with CA synthesis, so the relative expression levels of genes in the CA biosynthetic gene cluster, as well as of homologous genes needed for CA biosynthesis in the paralog and clavam gene clusters, were analyzed by RT-qPCR assays. The mycelium of F613-1 and Δ*cagRS* cultured at 24, 72, 120, 168, and 216 h was harvested, and the relative expression levels of genes involved in CA biosynthesis during the CA fermentation process were monitored. As shown in Figure 7, the expression level of *claR, oat2, oppA1, oppA2, car, cyp450, orf12, orf14, orf16*, and *gcaS* in the CA biosynthetic gene cluster increased during the fermentation process and peaked at 168 h; the expression level of *oat1* and *pah1* in the paralog gene cluster increased during the fermentation process and peaked at 168 h; and the expression level of *cas1* in the clavam gene cluster also increased during the fermentation process and peaked at 168 h. When compared with F613-1 levels, the expression levels of almost all of the 20 tested genes were decreased in Δ*cagRS* before the 120 h time point. This trend in gene expression was similar to the trend in the CA fermentation levels (Figure 3).

## Discussion

### *cagRS* Is a Global Regulatory TCS

Two-component regulatory systems constitute a family of proteins that mediate adaptation to changing environments by modifying the phosphorylated state of a pair of proteins: a sensor histidine kinase and a response regulator. TCSs are reported to be involved in a variety of bacterial cellular responses, such as biofilm development, morphological development, chemotaxis, sporulation, photosynthesis, osmoregulation, antibiotic production, and pathogenicity (Ogura and Tanaka, 2002; Bijlsma and Groisman, 2003; Hutchings et al., 2004; Mikkelsen et al., 2011). TCSs are very abundant in *Streptomyces* species and are reported to affect antibiotic production such as pimaricin biosynthesis (Mendes et al., 2007). The TCS orf22/orf23 of *S. clavuligerus* ATCC 27064 was reported to have high similarity to the TCS SCO4020/4021 of *S. coelicolor* A3(2) and to affect CA production in *S. clavuligerus* ATCC 27064; orf22/orf23 is located downstream of the CA gene cluster, and an *orf23* deletion mutant reduced CA production, cell growth and sporulation in strain ATCC 27064 (Song et al., 2009). However, Jnawali et al. (2008) reported that *orf23* deletion reduced CA production but had no effect on cell growth or morphological development. Beyond that, the mechanisms by which orf22/orf23 affects CA production have not been further investigated (Song et al., 2009). The TCS CagRS in *S. clavuligerus* F613-1, which was annotated as orf22/orf23 in ATCC 27064, is also located next to the CA biosynthetic gene cluster, and we found that deletion of CagRS significantly reduced CA production but did not affect the phenotype. We also found that single-gene deletion mutants of CagRS also significantly reduced CA production but did not affect the phenotype (Supplementary Figure S4). Our results with the *cagRS* double-gene deletion mutant were similar to those obtained with the previously described *orf23* deletion mutant (Jnawali et al., 2008).

In our ChIP-seq experiments, enriched peaks were concentrated near transcriptional start sites, indicating that the response regulator CagR is indeed a transcriptional regulatory protein. Both the transcriptome and ChIP-seq data revealed that the TCS CagRS mainly regulates genes involved in fatty acid degradation, G3P and arginine metabolism, and CA production. Given the phenotype of the mutant and our transcriptome and ChIP-seq results, we conclude that CagRS is a global regulatory TCS and that this system regulates aspects of both primary metabolism (such as G3P and arginine metabolism) and secondary metabolism such as CA production. However, this TCS has little or no effect on phenotype or spore development under the test conditions.

### *cagRS* Negatively Regulates Arginine Biosynthesis

As a direct precursor of CA biosynthesis, arginine is very important for CA production (Brian et al., 1993; Khaleeli et al., 1999). The arginine biosynthetic gene cluster of *S. clavuligerus* was characterized by Rodriguez-Garcia et al. (2000), and the schematic diagram of this cluster in *S. clavuligerus* F613-1 is shown in Figure 5A. In our study, transcriptome data revealed that deletion of *cagRS* resulted in the increased expression of the arginine biosynthetic gene cluster, and these findings were largely supported by RT-qPCR data for *argB, argC, argJ, argH*, and *argG*. Additionally, EMSAs revealed that CagR could bind to the promoter regions for *argG* and *argC*. Overall, these data suggest that CagRS modulates the biosynthesis of arginine in a negative manner.

### *cagRS* May Positively Regulate G3P Metabolism

**FIGURE 8:**
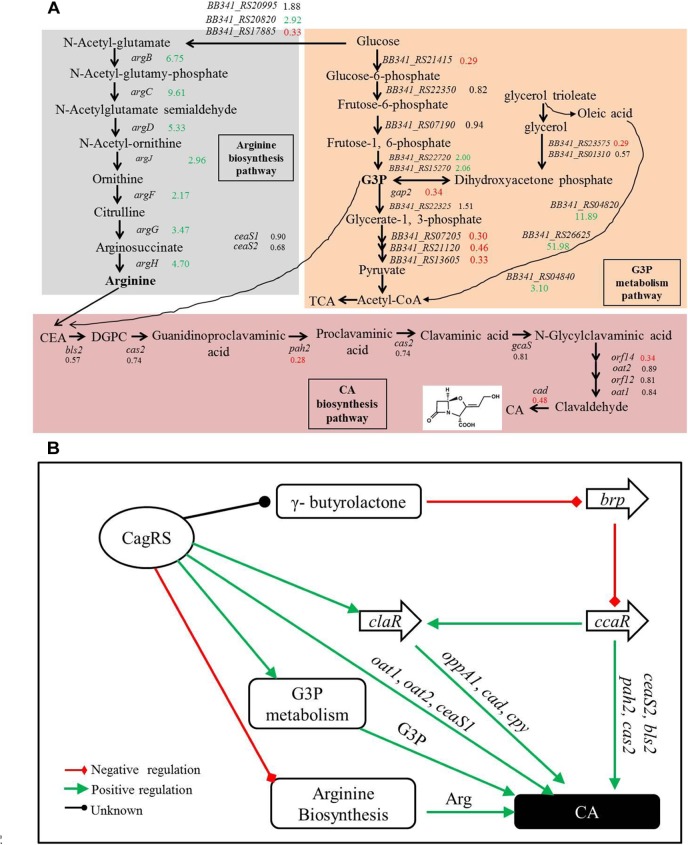
Clavulanic acid biosynthesis is regulated by the TCS CagRS. **(A)** Schematic overview of the expression profiles of genes involved in CA, G3P and the arginine metabolism pathway. CEA, *N*^2^-(2-carboxyethyl)-arginine; DGPC, deoxyguanidino-proclavaminic acid; TCA, tricarboxylic acid cycle. The numbers are the ratios of the comparative expression levels in Δ*cagRS* compared with the control F613-1, and the data are from the transcriptome and the RT-qPCR assays. Red indicates downregulation, green indicates upregulation, and black indicates no notable change. **(B)** Model of the TCS CagRS regulatory network of CA biosynthesis.

Glycerol, glycerol trioleate and other oils were reported to enhance CA production in *S. clavuligerus* (Baggaley et al., 1997; Ives and Bushell, 1997; Kim et al., 2009). The glycerol trioleate in the fermentation medium was absorbed into the cell and then was enzymatically dissociated into glycerol and oleic acid, with the produced glycerol converted into G3P (through primary metabolic pathways) for CA production. The produced G3P can also be converted into 1,3-bisphosphoglycerate by GAPDH in the glycolytic pathway and then enters the tricarboxylic acid cycle through pyruvate. Tricarboxylic acid cycle-intermediate accumulation is also reported to be associated with CA biosynthesis in *S. clavuligerus* (Ramirez-Malule et al., 2018). In this study, we found that conversion of glycerol into G3P may be inhibited in the Δ*cagRS* mutant strain because the gene (*BB341_RS23575*) that encodes glycerol-3-phosphate dehydrogenase was significantly down-regulated. Two genes (*gap1* and *gap2*), whose protein products are distinct GAPDHs, were characterized in *S. clavuligerus* 27064, and whereas the *gap1* mutant had twice the normal production levels of CA, the *gap2* mutant produced a level of CA similar to that of the wild-type strain (Li and Townsend, 2006), indicating that *gap1* plays a major role in converting G3P in *S. clavuligerus*. In Δ*cagRS*, the expression level of *gap2* was reduced significantly compared with F613-1 levels; however, the expression level of *gap1* was similar in the two strains, suggesting that the rate for conversion of G3P into acetyl-CoA in Δ*cagRS* would not differ greatly from that of F613-1. Additionally, our transcriptome and RT-qPCR analysis revealed that many genes involved in G3P and glycerol metabolism were significantly down-regulated in Δ*cagRS* compared with F613-1 (Table 1 and Figure 8A). Based on these data, we predict that the G3P concentration was therefore reduced, leading to the reduced CA production. Data from CA fermentation confirmedour hypothesis, as the amount of CA peaked at 168 h during the fermentation process whether supplemented with glycerol trioleate or not, but the CA concentration dropped significantly without glycerol trioleate.

### *cagRS* Positively Regulates the CA Biosynthetic Gene Cluster

Clavulanic acid is a potent β-lactamase inhibitor produced by *S. clavuligerus* F613-1 (Qin et al., 2017), a strain that also synthesizes cephamycin C (Reading and Cole, 1977) and a few other clavam-based structures. The biosynthesis pathway of CA and its related by-product (clavam) has been largely elucidated (Liras et al., 2008). However, there have been no reports on the global regulation of CA biosynthesis. In this study, deletion of CagRS resulted in markedly decreased production of CA, consistent with the reduced expression of multiple genes involved in CA biosynthesis at early time points, including *pah2, bls2, claR, car* (these four genes located in CA biosynthetic gene cluster), and *oat1* (in paralog gene cluster), compared with levels in F613-1. ClaR is a pathway-specific regulatory factor of CA biosynthesis and positively regulates the expression of *ceaS2* (Gomez-Escribano et al., 2006); the patterns of expression of the late CA synthetic genes, such as *orf12, orf14, oppA1, oppA2, orf16, gcaS* and *car*, were similar to *claR* expression patterns, indicating that *cagRS* may indirectly regulate these genes through *claR*. The above data indicate that CagRS positively regulates the expression of the CA biosynthetic gene cluster.

Our EMSAs revealed that CagR can interact with the promoters of *ceaS1, oat1*, and *oat2*. Both EMSA and ChIP-seq assays also revealed that CagR can interact with the *claR* promoter, indicating that CagR can directly regulate *claR*, thereby affecting the expression of ClaR target genes in the CA biosynthetic pathway. Given the above data, we propose that CagRS modulates CA biosynthesis through *claR, ceaS1, oat1*, and *oat2.* CcaR is another pathway-specific regulatory factor involved in the biosynthesis of CA and was reported to positively regulate the expression of early stage CA biosynthetic genes (such as *ceaS2, bls2, pah2*, and *cas2*) and *claR* (Santamarta et al., 2002, 2011; Alvarez-Alvarez et al., 2014), suggesting that CagR and CcaR both directly regulate CA synthesis. In addition, the γ-butyrolactone signaling system negatively regulates CA production through inhibiting *ccaR* (Santamarta et al., 2005). Interestingly, our ChIP-seq assays revealed that CagR can bind the promoter of the *BB341_RS25520* (*avaA2*) gene, which encodes a gamma-butyrolactone biosynthesis protein, suggesting that CagR can also regulate CA production through the γ-butyrolactone signaling system-CcaR pathway (Figure 8B).

In conclusion, we found that CagR can modulate expression of the CA biosynthetic gene cluster and also affect genes involved in the metabolism of G3P and arginine, two direct precursors of CA. Our results provide new insights into the global regulation of CA biosynthesis and provide an important resource for future metabolic engineering efforts for CA production in *S. clavuligerus*.

## Author Contributions

GC designed the work. RQ executed the experiments. JF analyzed the data and prepared the manuscript draft and revised the manuscript. GZ and CL carried out the interpretation of data, drawing up figures and statistical analysis. NK helped in data analysis. CZ contributed to the experimental design, manuscript preparation, and submission. All authors read and approved the final manuscript.

## Conflict of Interest Statement

The authors declare that the research was conducted in the absence of any commercial or financial relationships that could be construed as a potential conflict of interest.

## Footnotes

1 http://www.genoscope.cns.fr/fastxtend

2 http://hannonlab.cshl.edu/fastx_toolkit/

3 http://www.ncbi.nlm.nih.gov/geo

4 http://amigo.geneontology.org

5 https://www.genome.jp/kegg/pathway.html
