# Supplementary material for: The CagRS Two-Component System Regulates Clavulanic Acid Metabolism via Multiple Pathways in Streptomyces clavuligerus F613-1
Source: Front Microbiol. 2019 Feb 14;10:244. doi: 10.3389/fmicb.2019.00244 (PMC6382702; doi:10.3389/fmicb.2019.00244)
Supplement: Supplementary file 1 [file Data_Sheet_1.doc]

**Fu, et al., The CagRS two-component system regulates clavulanic acid metabolism via multiple pathways in *Streptomyces clavuligerus* F613-1**

**Supplementary Material**

**Table S1 Plasmids and strains used in this study.**

| **Strains or plasmids** | | | **Description** | **Source/Reference** |
| --- | --- | --- | --- | --- |
| ***Streptomyces clavuligerus* strains** | | | | |
| F613-1 | | Industrial clavulanic acid producer | |  |
| △*cagRS* | | F613-1 mutant with *cagRS* double-gene deletion | | This study |
| △*cagRScom* | | △*cagRS* complemented with *cagRS* | | This study |
| △*cagRScom*-pSET152 | | △*cagRS* complemented with the empty vector pSET15 | | This study |
| cagR-Flag | | △*cagRS* complemented with the fusion fragment *cagR*-Flag-*cagS* based on pSET152 plasmid; △*cagRS*:: *cagR*-[Gly_4_Ser]_3_-3*Flag-*cagS* | | This study |
| ***E. coli* strains** | | | | |
| DH5α | General cloning host | | | Trans (China) |
| BL21 (DE3) | Strain used for protein expression | | | Trans (China) |
| *E. coli* MA18 | *E. coli* containing pJTU1728 vector, *bla*^a^ | | |  |
| ET12567/pUZ8002 | Strain used for conjugation between *E. coli* and *Streptomyces spp* | | |  |
| **Plasmids** |  | | |  |
| pJTU1278 | *E. coli*-*Streptomyces* shuttle vector, *tsr*^a^ *bla*^a^ *oriT* | | |  |
| pSET152 | *Streptomyces* integrated vector | | | BioVector (China) |
| pET-15b | Expression vector containing the T7 promoter and 6×His-thrombin | | | Novagene (USA) |
| pSET-cagRS | pSET152 containing the coding sequence of *cagRS* plus *cagR* upstream intergenic sequence | | | This study |
| pET-cagR | pET15b containing the coding sequence of *cagR* | | | This study |
| pEasy-Blunt-Simple | General cloning vector | | | Trans (China) |
| pJTU-cagRS | pJTU1278 containing the *cagRS*-disrupted cassette | | | This study |
| pSET-cagRFlag | pSET152 containing the fusion fragment *cagS*-Flag-*cagR* | | | This study |

^a^ *tsr,* thiostrepton-resistance gene; *bla*, ampicillin-resistance gene.

**Table S2 Primers used in this study.**

| **Oligonucleotides** | **DNA Sequence (5’→3’)*** |
| --- | --- |
| **Recombinant plasmid pJTU-cagRS construction** | |
| cagRS L-F | GGACTAGTGACGGTCGGCAGAACGGCAA |
| cagRS L-R | CCGGATCCAATGCAAGCTTAGACCACCGACACCACCG |
| cagRS R-F | GGAAGCTTGTGGTCCATGCGGGGTCT |
| cagRS R-R | CGGGATCCCCCATTGAACCAACCCCG |
| **Validation of △*cagRS* knockout and △*cagRS*com strain** | |
| cagRScom V-F | GCGGATCATGCGGGCGGAGC |
| cagRScom V-R | TGGCCGCCGTCGTCGTGGAG |
| **Recombinant plasmid pSET-cagRS construction** | |
| cagRScom-F | AAGAAGGGAGCGGACATATGACCCGGATGGCCCCCGAGACC |
| cagRScom-R | GGCTGCAGGTCGACTCTAGACTAGGCCGTCCCCGTCCCCGC |
| **Recombinant plasmid pET-cagR construction** | |
| cagRHis-F | CATATGACCCGGATGGCCCCCGAGACCCCGCATGGA |
| cagRHis-R | CTCGAGTCATGTCCCCTCCGCGGGCATCAGCAGATA |
| **Recombinant plasmid pSET-cagRFlag construction** | |
| cagRFlag-F1 | ggctgcaggtcgactctagaGCGGATCATGCGGGCGGAGC |
| cagRFlag-R1 | tggcggtagtgactacaaagaccatgacggtgattataaagatcatgacatcgattacaaggatgacgatgacaagACATGAGACGGAGGTGGGCCGG |
| cagRFlag-F2 | CtttgtagtcactaccgccaccgccagagccacctccgcctgaaccgcctccaccCCCCTCCGCGGGCATCAGCAG |
| cagRFlag-R2 | tcgcgcgcggccgcggatccTGGCCGCCGTCGTCGTGGAG |
| **EMSAs** | |
| argG p For | CGACGCGCTCGGTCACAGGA |
| argG p Rev | GGCCTGACCGGCCCTCCG |
| argB p For | GGCCTTGGGGAGGGCGTTGT |
| argB p Rev | GCTCGTGACCTCGACGGCGG |
| argC p For | GCAACCGCCACCCGTACCGT |
| argC p Rev | CCACCCCACCGGCCACTGAC |
| argJ p For | TTCACCACCAGTGCCAGATC |
| argJ p For | GATCGACAACCTGACCAAGG |
| argH p Rev | AAGTGCACCGAGGCGGAGAG |
| argH p Rev | TCGCCGCCAAGCGCGACCTG |
| oat2 p For | TGCCGTGCGCCGCCCGTGGGTGCTGTACAG |
| oat2 p Rev | GCGTCTTCGGTGTGCTGTCGGACATGTCAG |
| oat1 p For | CGTTCCCGTTCCAGCGCGCTGTCGAGCCGC |
| oat1 p Rev | CCCTGATCCCGGCGTGACCGTGGTGCAC |
| ceaS1 p For | GACGATGGACGCCATGCCTTGAACGCGCCT |
| ceaS1 p Rev | GGCTTTCGCGGTCGTGGTGGCCATGGAGATCT |
| claR p For | TTCCCGGGACGGCGCCCGGAG |
| claR p Rev | GAGGTCTCCGCAGTGTCCCAC |
| ceaS2 p For | CACGCACCGTCACTCTTCGTCCCCTCTTCA |
| ceaS2 p Rev | AAACACCTTCCCCACACCGTGATCACGCAA |
| pah2 p For | CTAGTCCCGCCACGGGGAGCCCGCCGGACG |
| pah2 p Rev | TGCGAGTCGATGCGCTCCACGCTTTCAGCT |
| oppA1 p For | GTTCCCTGTCCGCTCCCGTCCCGTGTGGTT |
| oppA1 p Rev | GTCGTCGACCGAGTGGTCTCCATGTCCCTC |
| cad p For | GAGAGCGCCGCGGTATTCACCATTCCCC |
| cad p Rev | TGGAGTGCGGATGGCATCATTTCCTCCAC |
| cyp450 p For | GAATTCCCTTCGGTGGACCGGGTTATGACT |
| cyp450 p Rev | CTGAGGCGCTGCCTCGTTCATCATCCACC |
| orf12 p For | AATCAGCTTTCTTCATCATCGCTCCTTG |
| orf12 p Rev | TCGTCAAAATCCCGTCTGCGTCGCGAC |
| orf14 p For | TCGCCGAAGAAGACTAGGTCCATCACC |
| orf14 p Rev | CTCCAGTTCGCCGGCGGTGTCGTTCATGCG |
| gcaS p For | CTTTCCGACGACGATGGCTCCCACCCCCCGA |
| gcaS p Rev | CCAGTTGACCGCGCCGATCACGTTCGGGAC |
| cas1 p For | TGGTGTGGTTCCTTTCCTTGGTTCCTC |
| cas1 p Rev | CGCTCGCTGCAAGAGGAACATCCACAG |
| pah1 p For | CAGGAACGGCAGGAGCGGTCTCGGAGG |
| pah1 p Rev | GAGACGGCGGTGGACACCCTGGCCGTT |
| ceaS1 p For | GACGATGGACGCCATGCCTTGAACG |
| ceaS1 p Rev | GCTTTCGCGGTCGTGGTGGCCATGGAG |
| **RT-qPCR** | |
| 16S-RT For | GAGATCCGCCTTCGCCACCG |
| 16S-RT Rev | CTGCATTCGATACGGGCAGGC |
| ceaS2-RT For | AGGCCGCGTCGATTCTCTTCG |
| ceaS2-RT Rev | AGAGGTTGGTCATACCGGGGC |
| oat2-RT For | CGACTTCACCGTCCTCGCCT |
| oat2-RT Rev | GGTCGCGACATTCGCGTTGC |
| claR-RT For | TGCTGTCGCTGGTCTCCACG |
| claR-RT Rev | TAGGCCGCGTCCACCTGGTA |
| cad-RT For | CCGACTGGACCCGGATGATCG |
| cad-RT Rev | TTCGTGGCCTGGTAGACGGC |
| orf12-RT For | AGGGCCGACAAGGAGCGATG |
| orf12-RT Rev | GTCCGGACGAGGTCAGCAGC |
| orf14-RT For | CGGCGAACGACGACGAAACG |
| orf14-RT Rev | CCAGTCGTCGAGGGCGGTC |
| gcas-RT For | CACCCCTGGCCGACTATGCC |
| gcas-RT Rev | GCCCGTGGGTGTACCAGGAC |
| cas1-RT For | GAGGACCGCTCCCTGCTGAC |
| cas1-RT Rev | CTCCGAGGACAGGTGGTGCG |
| ceaS1-RT For | CACGACCGCGAAAGCCATGC |
| ceaS1-RT Rev | GTGAACTCGTGCCGGGTCAGG |
| oat1-RT For | TGCTGTCCGGCAACGCCAAC |
| oat1-RT Rev | ACACGGGACATCGGATACCGC |
| RS18990-RT For | CCTGCGGGTGAGTTCCTCCAG |
| RS18990-RT Rev | TCTCCGAACTCCCCAAGGCGT |
| RS13605-RT For | GCTCCGCGACGACGACTATG |
| RS13605-RT Rev | TCCCAGCCACCGTGGTTCAC |
| RS18805-RT For | TATAGGCGGCCGTGTTGCCG |
| RS18805-RT Rev | CCTACCCGGTGGTCGAGAGC |
| RS13595-RT For | GGCCGAGATCCTCAGCTGGT |
| RS13595-RT Rev | CCGTCATAGGGGATCGGCAGC |
| RS04820-RT For | ACCCTGGTCTGTCTGACCGC |
| RS04820-RT Rev | GTCCGTCATGCCGAGGGAGG |
| RS04840-RT For | CCTTTCCCAGCTCCGCGACG |
| RS04840-RT Rev | TGAGCGCGTTGAGCCGTTCC |
| argC-RT For | CCTGCGAGAACGCCGTCAGC |
| argC-RT Rev | GACGGTACGGGTGGCGGTTG |
| bls2-RT For | TGCCGCTGTACACCTGTGTGG |
| bls2-RT Rev | CGCGGGCACCTGGTAGACAC |
| pah2-RT For | ACGGCGCAGAGCCATCTGTC |
| pah2-RT Rev | TTGGTGTCGGAGTGCGCGTC |
| cas2-RT For | CTCCGAGCTTCCCGAGGTGC |
| cas2-RT Rev | CGCGCAGCAGCAGATAACCG |
| oppA1-RT For | CGCCGGTCCGACCTACTTCC |
| oppA1-RT Rev | GATCCATCCCCGCGAACGGC |
| cyp450-RT For | AGCCAGGTGTGGCTGGTGAC |
| cyp450-RT Rev | GCGGATGAACGACGCCGACT |
| fd-RT For | GCCCCCGAGATCTTCGACCAG |
| fd-RT Rev | TAGCCCTCGGTGACCGTGAT |
| orf13-RT For | TCCTCTCCGCGATGCGGTTC |
| orf13-RT Rev | GCATGCCGATGTCGATGGCG |
| oppA2-RT For | ACGTCTGGGTGTGGCTGCTC |
| oppA2-RT Rev | GCAGCCGGTAGGTCCAGGTC |
| orf16-RT For | CACCGTCTGCTTCCCGCACG |
| orf16-RT Rev | GCGGTGCTTGGTCATGTCGG |
| pah1-RT For | GCAGGAGCGGTCTCGGAGG |
| pah1-RT Rev | GCTCCCGTTCCCGTACCGAC |

* Restriction enzyme sites are underlined and were used for cloning purposes. Bases corresponding to the triple-Flag tag are shown in red lowercase letters. Bases corresponding to the [Gly_4_Ser]_3_ linker are shown in green lowercase letters.

Figure S1


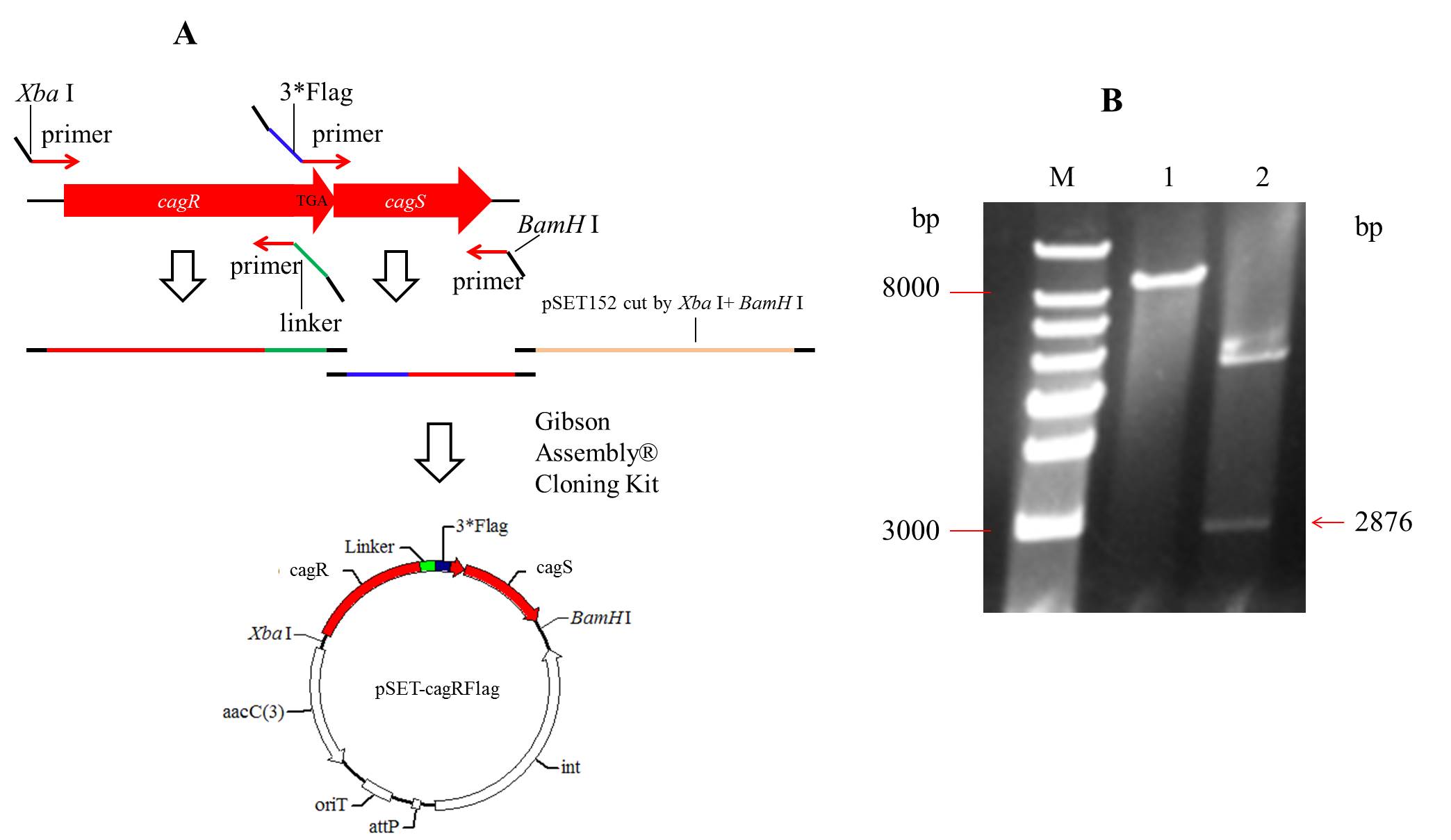


**Fig. S1** Construction of the recombinant plasmid pSET-cagRFlag. (A) Flow chart for construction of pSET-cagRFlag*.* The black lines in the primers indicate the corresponding overlapping sequences for joining two different fragments together, green lines indicate the [Gly_4_Ser]_3_ linker sequence (GGGGSGGGGSGGGGS), and red lines indicate the 3*Flag tag sequence (DYKDHDGDYKDHDIDYKDDDDK). (B) PCR verification of pSET-cagRFlag. M, DNA ladder marker; lane 1, *Xba*I digestion; lane 2, *Xba*I + *BamH*I double digestion.

Figure S2


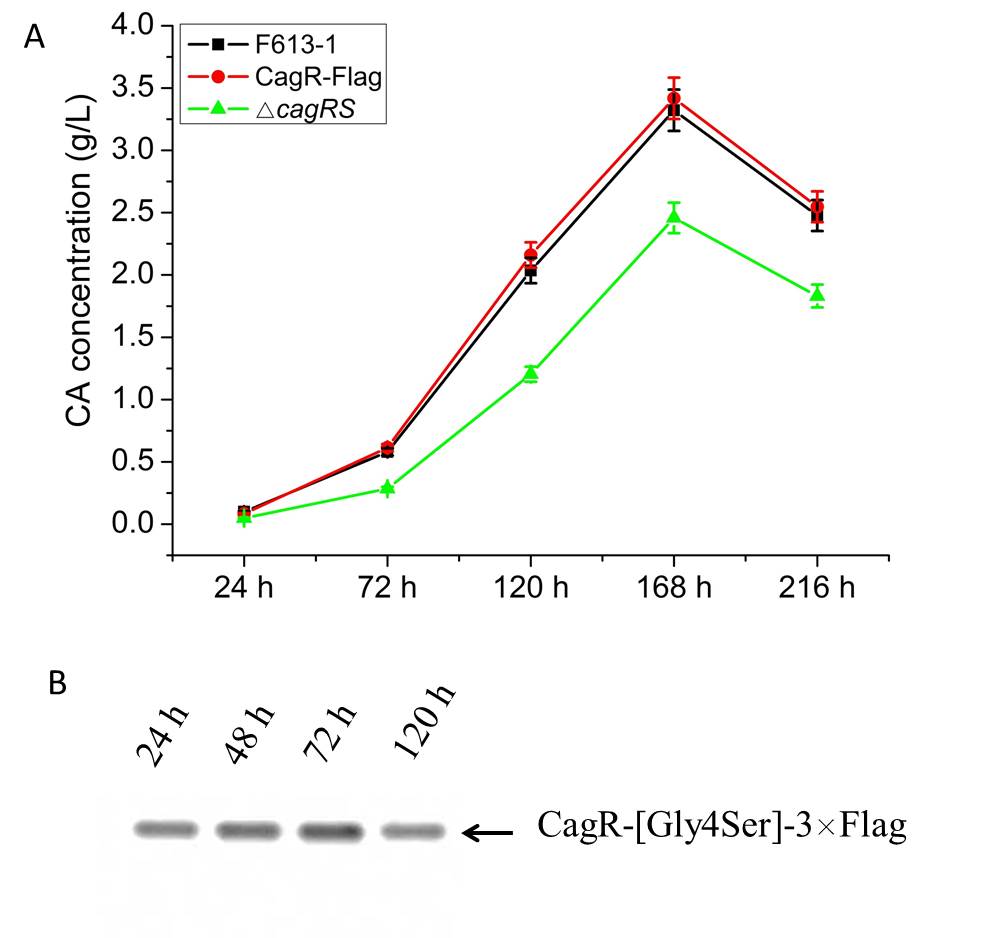


**Fig. S2** (A) CA liquid fermentation titers of strains F613-1, cagR-Flag and △*cagRS* in SCF fermentation medium*.* (B) Western blot detection of triple FLAG-tagged CagR using anti-Flag antibody.

Figure S3


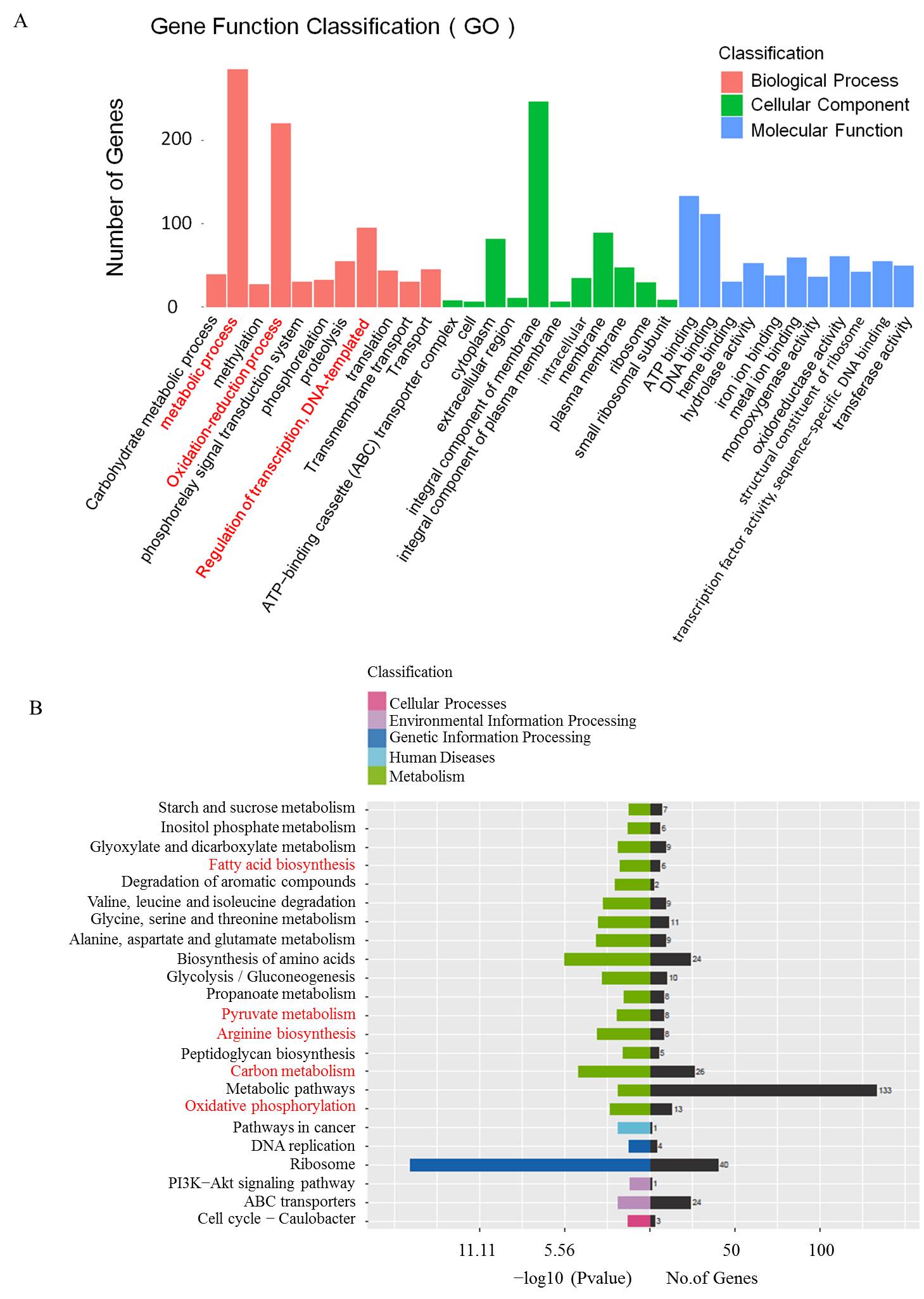


**Fig. S3** Transcriptome analysis of differentially expressed genes in △*cagRS* compared with F613-1. (A) GO term enrichment of genes with significantly changed expression (log2 FC>1) in △*cagRS* (compared with F613-1). (B) KEGG clustering analysis of genes with significantly changed expression (log2 FC>1) in △*cagRS* (compared with F613-1).

Figure S4


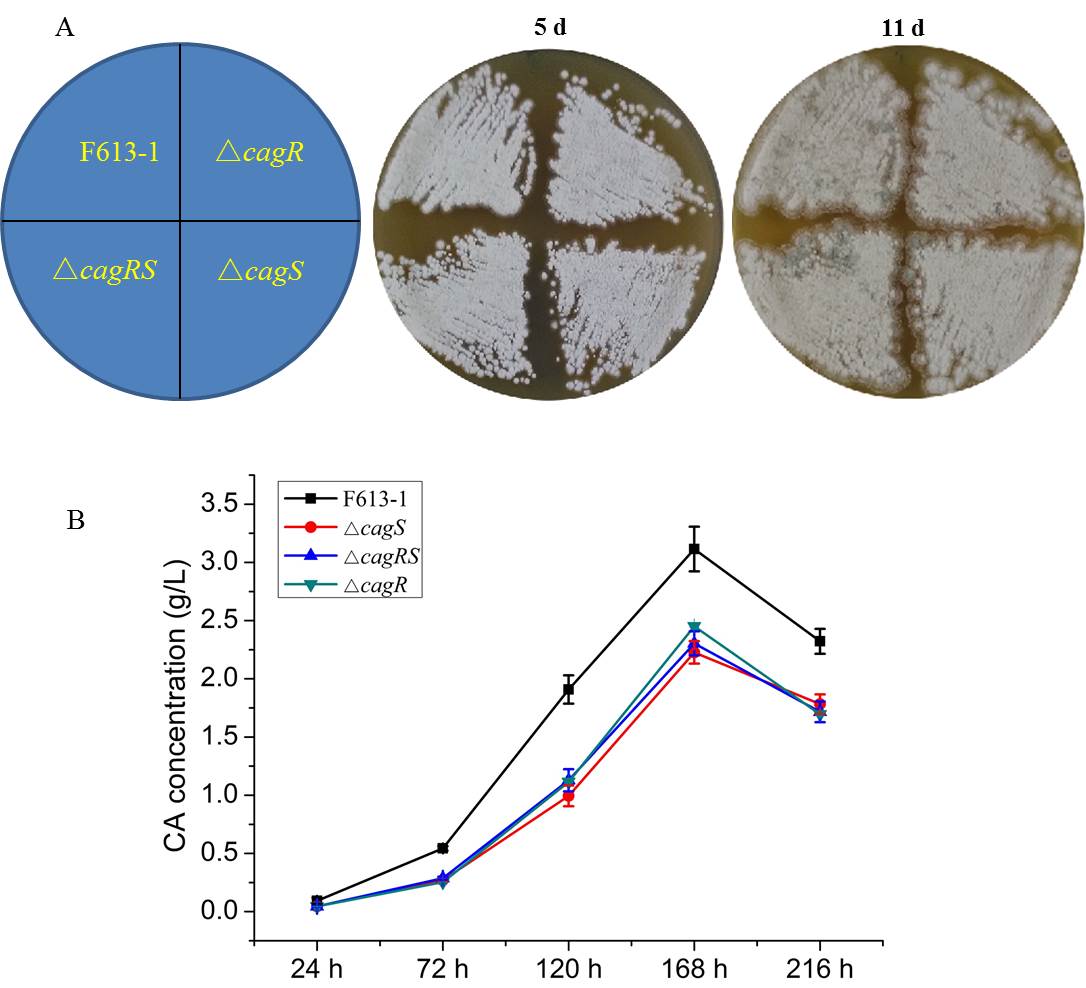


**Fig. S4** (A) Phenotype of F613-1, △*cagRS*, △*cagR* and △*cagS* on BCSA solid medium. (B) CA liquid fermentation titers of strains F613-1, △*cagRS*, △*cagR* and △*cagS* in SCF fermentation medium supplemented with 1.6% (v/v) glycerol trioleate*.*

Figure S5


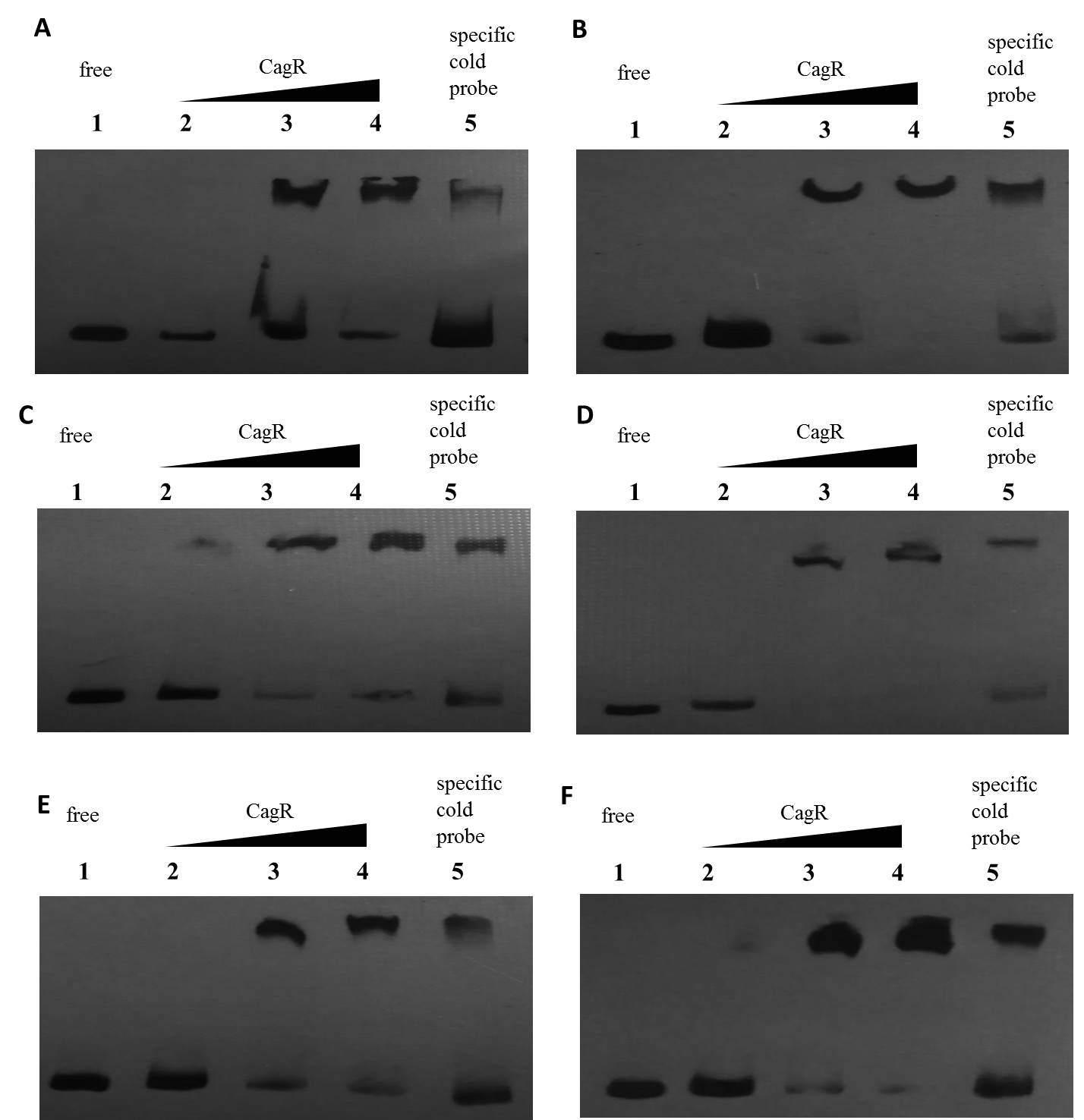


**Fig. S5** Specific binding of CagR to the target promoters. A fixed amount of probe was incubated with no CagR (Lane 1); 0.75-3.0 μg CagR (Lanes 2-4); 3.0 μg CagR , and 100-fold excess of unlabeled specific probe (Lane 5). The appropriate amount of polydI/dC (1.0 mg) was used as competitor. (A) 299 bp of the *argG* upstream region; (B) 160 bp of the *argC* upstream region; (C) 181 bp of the *claR* upstream region; (D) 214 bp of the *ceaS1* upstream region; (E) 169 bp of the *oat2* upstream region; (F) 171 bp of the *oat1* upstream region.

**References：**

He, Y., Wang, Z., Bai, L., Liang, J., Zhou, X., and Deng, Z. (2010). Two pHZ1358-derivative vectors for efficient gene knockout in *streptomyces.* *J Microbiol Biotechnol* 20**,** 678-682.

Jin, X., Cao, G., Zhang, X., Chen, Y., Wang, L., and Zhong, C. (2015). Studies on the formation and synthetic mechanism of related substance G in potassium clavulanate production. *Brazilian Journal of Pharmaceutical Sciences* 51**,** 77-83. doi:10.1590/s1984-82502015000100008

Kieser, T., Bibb, M.J., Buttner, M.J., Chater, K.F., and Hopwood, D.A. (2000). Practical *Streptomyces* Genetics. *John Innes Foundation, Norwich, England*.

Qin, R., Zhong, C., Zong, G., Fu, J., Pang, X., and Cao, G. (2017). Improvement of clavulanic acid production in *Streptomyces clavuligerus* F613-1 by using a claR - neo reporter strategy. *Electronic Journal of Biotechnology* 28**,** 41-46. doi:10.1016/j.ejbt.2017.05.002
